# FUT8 reprograms glycolytic metabolism to promote PKM2 lactylation and drive clear cell renal cell carcinoma progression

**DOI:** 10.1038/s41420-026-03013-1

**Published:** 2026-03-19

**Authors:** Zikai Guo, Hongxiao Jiang, Xu Wang, Ke Xuan, Huidong Zhong, Chengxi Liu, Mengkai Zhang, Zhichao Li, Weiren Huang, Yangyang Sun

**Affiliations:** 1 Department of Urology, The First Affiliated Hospital of Anhui Medical University, Hefei, China; 2 The First Affiliated Hospital, Shenzhen University; Shenzhen Second People’s Hospital; Medical Innovation Technology Transformation Center of Shenzhen Second People’s Hospital, Shenzhen, China; 3 Guangxi University of Chinese Medicine, Nanning, China; 4 The Third Affiliated Hospital of Anhui Medical University (The First People’s Hospital of Hefei), Hefei, China; 5 Department of Oncology, Ji’an Central Hospital, Ji’an, China; 6 Department of Applied Biology and Chemical Technology, Food Safety and Technology Research Centre, and Research Centre for Chinese Medicine Innovation, The Hong Kong Polytechnic University, Hung Hom, Kowloon, Hong Kong SAR, China; 7 State Key Laboratory of Quantitative Synthetic Biology, Shenzhen Institute of Synthetic Biology, Shenzhen Institutes of Advanced Technology, Chinese Academy of Sciences, Shenzhen, China; 8 Guangdong Key Laboratory of Systems Biology and Synthetic Biology for Urogenital Tumors, Shenzhen, China; 9 GuangDong Engineering Technology Research Center for Clinical Application Of Cancer Genome, Shenzhen, China

**Keywords:** Cancer metabolism, Renal cell carcinoma

## Abstract

Clear cell renal cell carcinoma (ccRCC) is characterized by the loss of the von Hippel–Lindau (VHL) gene, leading to constitutive activation of hypoxia-inducible transcription factors (HIFs) and metabolic reprogramming toward aerobic glycolysis. Although core fucosylation catalysed by fucosyltransferase 8 (FUT8) is known to regulate receptor signaling and tumor malignancy, its role in metabolic regulation of ccRCC remains poorly defined. Here, we demonstrate that FUT8 knockdown significantly suppresses ccRCC proliferation and migration both in vitro and in vivo. Mechanistically, FUT8 enhances HIF-1α–driven glycolysis, increasing lactate production and promoting pan-lysine lactylation (pan-Kla). Specifically, FUT8 promotes pyruvate kinase M2 (PKM2) K115 lactylation, which boosts its enzymatic activity while reducing nuclear localization, thereby driving epithelial–mesenchymal transition and malignant progression. Collectively, our findings reveal the FUT8–HIF-1α–lactate–PKM2 axis as a key mechanism that links core fucosylation to metabolic reprogramming and malignant progression in ccRCC and highlights FUT8 as a promising therapeutic target.

## Introduction

Kidney cancer accounted for 434,419 new cases and 155,702 deaths worldwide in 2022 [1]. Among these, renal cell carcinoma (RCC) constitutes about 90% of cases, with clear cell RCC (ccRCC) representing the predominant histological subtype (about 75%) [2, 3]. A molecular hallmark of ccRCC is inactivation of the von Hippel–Lindau (VHL) tumor suppressor gene, observed in approximately 80%–90% of sporadic cases [4]. VHL loss stabilizes hypoxia-inducible factors (HIFs), leading to constitutive activation of hypoxia-responsive pathways and driving metabolic reprogramming, tumor progression, and therapeutic resistance [5].

Clear cell renal cell carcinoma is a metabolically dysregulated tumor [6, 7], rendering metabolic reprogramming an appealing therapeutic vulnerability [8]. Its canonical metabolic hallmarks include enhanced aerobic glycolysis and impaired mitochondrial oxidative phosphorylation [9–12], underscoring the centrality of glucose metabolism in tumor initiation and progression. This Warburg-like metabolic state is largely HIF-driven. In a mouse model, HIF-1α has been shown to induce glycolytic reprogramming and tumorigenesis [13]. A defining consequence of this metabolic shift is lactate accumulation. In addition to fueling an acidic tumor microenvironment that promotes angiogenesis, immune evasion, and metastasis [14–18], lactate also acts as a substrate for lysine lactylation (Kla), which is an acyl modification that directly links metabolic activity to epigenetic and post-translational regulation [19]. Since its discovery in 2019 [20], lactylation has been implicated in transcriptional control, immune modulation, and tumor progression [21–23]. Proteomic surveys further reveal widespread non-histone lactylation in cancer, with functional roles in metabolic regulation and oncogenesis [24, 25]. These findings underscore lactylation as a key mediator of tumor biology and highlight the need to define its upstream regulators.

Protein glycosylation represents another crucial regulatory layer in cancer. Core fucosylation, catalyzed exclusively by fucosyltransferase 8 (FUT8), modifies N-glycans at the innermost GlcNAc residue via an α1,6 linkage [26]. Elevated FUT8 expression has been documented in diverse malignancies—including lung, breast, prostate, liver, and colorectal cancers—as well as melanoma and head and neck squamous cell carcinoma [27–31]. Functionally, FUT8 enhances oncogenic signaling through its core fucosylation activity, which promotes ligand–receptor interactions and potentiates key pathways such as TGF-β and epithelial–mesenchymal transition (EMT) [28, 32]. FUT8 expression is itself regulated by oncogenic transcriptional networks, including a FUT8–STAT3 positive feedback loop [31] and lncRNA HOTAIR–p300–STAT3 complexes [33]. While these established mechanisms underscore the pivotal role of FUT8 in cancer progression through glycosylation-dependent signaling, its role in metabolic reprogramming remains largely unexplored. We propose that FUT8 may influence tumor progression not only through glycosylation but also by regulating lactate metabolism and lactylation, representing a significant extension of its functional scope.

Pyruvate kinase M2 (PKM2), a glycolytic enzyme highly relevant to cancer biology, catalyzes the final step of glycolysis and toggles between a tetrameric state with high enzymatic activity and a dimeric state with protein kinase activity [34, 35]. Post-translational modification (PTM) dictates this equilibrium: methylation and succinylation favor tetramerization, whereas phosphorylation and acetylation promote the dimeric state [36]. Recent evidence suggests that lactylation also regulates PKM2, with Lys62 modification enhancing enzymatic activity and influencing macrophage polarization [37], while inhibition of PKM2 lactylation promotes apoptosis and antitumor immunity [38]. These findings position PKM2 as a potential integrator of metabolic and PTM regulation in cancer.

Here, we demonstrate that FUT8 is upregulated in ccRCC and correlates with poor prognosis. Functionally, FUT8 drives HIF-1α-dependent glycolytic reprogramming, elevates lactate production, and promotes widespread Kla, including lactylation of PKM2 at lysine 115 (K115). Mechanistically, this modification alters PKM2 activity and subcellular localization, thereby enhancing EMT and tumor progression. Collectively, we define a FUT8–HIF-1α–lactate–PKM2 axis that couples metabolic reprogramming to PTM, nominating FUT8 and its lactylation pathway as candidate biomarkers and therapeutic targets in ccRCC.

## Results

### FUT8 is highly expressed in ccRCC and associated with poor prognosis

**Fig. 1:**
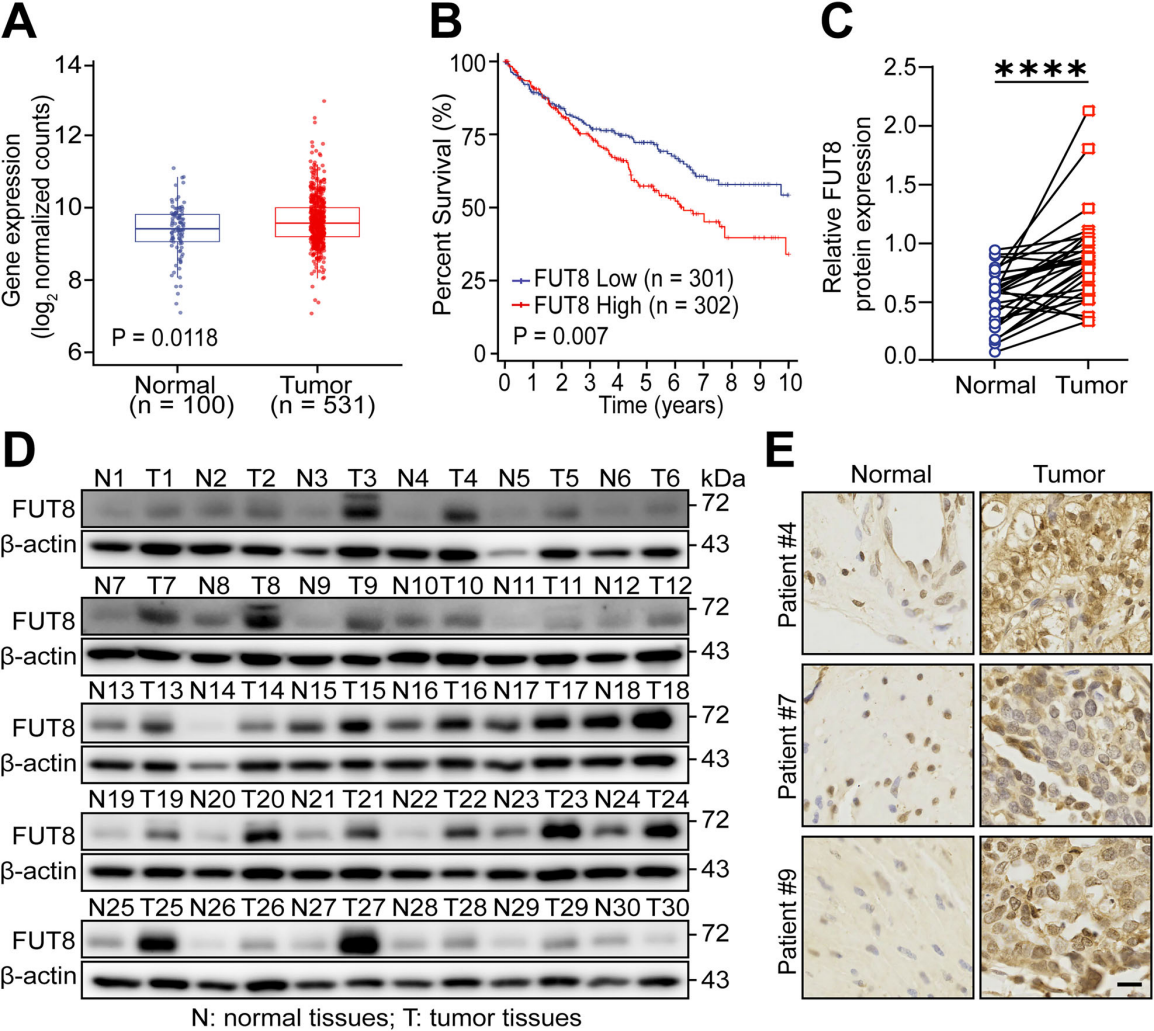
FUT8 is highly expressed in ccRCC and associated with poor prognosis. **A** FUT8 expression in ccRCC and normal kidney tissues from the TCGA-KIRC dataset. **B** Kaplan–Meier overall survival analysis comparing patients with low versus high FUT8 expression. **C**, **D** FUT8 protein levels in ccRCC tissues (T) and matched adjacent normal tissues (N) assessed by Western blotting (*n* = 30 patient pairs). **E** Representative immunohistochemistry images of FUT8 in ccRCC and normal tissues (Scale bar: 100 µm). Data are presented as mean ± SD. Statistical significance was evaluated using a paired t test. ns, *P* > 0.05; *P < 0.05; **P < 0.01; ***P < 0.001; ****P < 0.0001.

To evaluate the clinical significance of FUT8 in ccRCC, patients were stratified into high- and low- groups based on FUT8 levels. Kaplan–Meier analysis revealed a clear separation of overall-survival (OS) curves, with significantly shorter OS in the high-expression group, indicating that elevated FUT8 is associated with poor prognosis (Fig. 1B).

To characterise FUT8 expression in tumors, we compared mRNA levels between ccRCC tissues and normal kidney tissues using the TCGA and GTEx datasets. FUT8 expression was markedly higher in ccRCC, with a statistically significant difference relative to normal tissue (Fig. 1A). Because transcript abundance may not fully reflect protein dynamics, we performed orthogonal validation using independent paired clinical specimens. Western blotting of 30 paired ccRCC tumors and adjacent normal tissues demonstrated stronger FUT8 bands in tumors for the majority of cases (Fig. 1D), with densitometric quantification confirming a significant tumor-associated upregulation (Fig. 1C). This paired design minimizes inter-individual variability and enhances the robustness of the findings. At the histological level, immunohistochemistry further delineated the spatial distribution of FUT8. Representative sections demonstrated more intense staining in ccRCC than in normal kidney, with increases in both the proportion of positive cells and staining intensity (Fig. 1E). Together, these findings demonstrate that FUT8 is markedly upregulated in ccRCC at both transcript and protein levels and associates with adverse clinical outcomes, indicating its potential as a tumor biomarker for ccRCC.

### Knockdown of FUT8 suppresses ccRCC proliferation and migration in vitro and in vivo

**Fig. 2:**
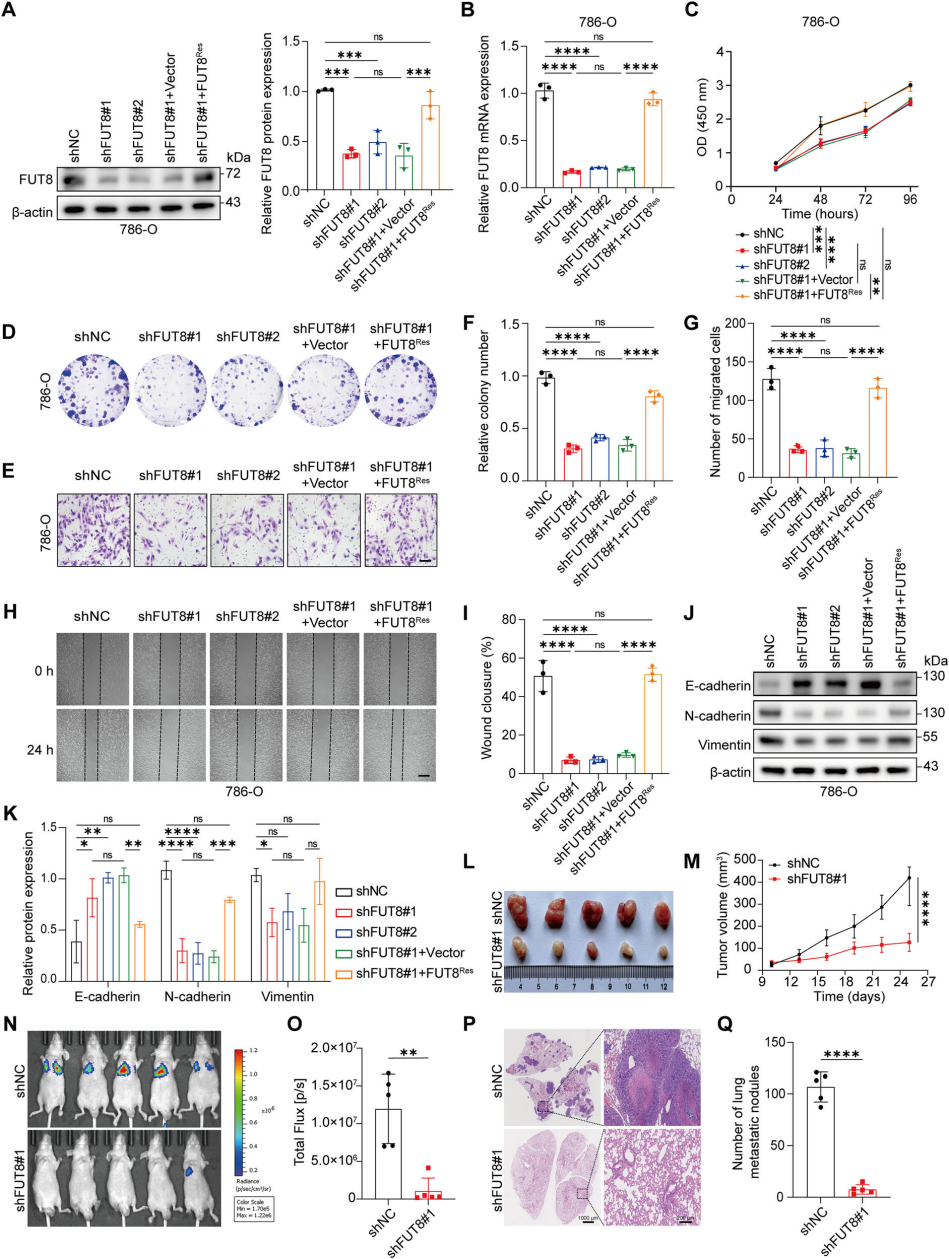
Knockdown of FUT8 suppresses ccRCC proliferation and migration in vitro and in vivo. **A**, **B** Verification of FUT8 knockdown and rescue efficiency in 786-O cells by Western blotting and qRT-PCR. For rescue experiments, shFUT8#1 cells were transduced with an shRNA-resistant FUT8 construct (FUT8^Res^), while Vector served as the matched empty control. **C**, **D**, **F** Cell proliferation of 786-O cells under the indicated conditions was assessed by CCK-8 and colony formation assays, with quantitative analysis of colony numbers. **E**, **G**–**I** Cell migration of 786-O cells under the indicated conditions was evaluated by Transwell assays (scale bar: 50 µm) and wound-healing assays (scale bar: 50 µm), with quantification of migrated cells and wound closure. **J**, **K** Western blot analysis and densitometric quantification of epithelial–mesenchymal transition (EMT) markers, including E-cadherin, N-cadherin, and vimentin, in control, FUT8-knockdown, and FUT8-rescued 786-O cells. **L**, **M** A subcutaneous xenograft model in BALB/c nude mice showing tumor growth over time (*n* = 5 per group). **N**–**Q** Lung metastasis of 786-O cells assessed by bioluminescence imaging, total flux quantification, hematoxylin and eosin (H&E) staining of lung sections (Scale bars: 1000 and 200 μm), and enumeration of metastatic nodules. Data are presented as mean ± SD. Statistical significance was assessed using unpaired t tests or one-way ANOVA as appropriate. All in vitro experiments were performed in three independent biological replicates (*n* = 3). ns, *P* > 0.05; *P < 0.05; **P < 0.01; ***P < 0.001; ****P < 0.0001.

To investigate the functional role of FUT8 in ccRCC, we silenced FUT8 expression using shRNA and systematically evaluated its effects on malignant phenotypes in vitro and in vivo. In 786-O cells, efficient FUT8 knockdown was confirmed by Western blotting and qRT**-**PCR, which showed a marked reduction in FUT8 protein and mRNA levels compared with negative controls (Fig. 2A–B). To assess on-target specificity, FUT8-knockdown cells were further transduced with an shRNA-resistant FUT8 construct (FUT8^Res^), which restored FUT8 expression to levels comparable to control cells (Fig. 2A–B). Consistent results were also observed in Caki-1 cells (Fig. S1A–B).

In cell-based functional assays, shRNA-mediated FUT8 depletion significantly suppressed the proliferative capacity of 786-O cells and Caki-1 cells. CCK-8 assays revealed reduced cell growth rates following FUT8 knockdown, whereas re-expression of shRNA-resistant FUT8 partially restored proliferative activity (Figs. 2C; S1C). Consistently, colony formation assays demonstrated that FUT8-knockdown cells formed fewer and smaller colonies, an effect that was substantially rescued by FUT8^Res^ re-expression (Figs. 2D, F; S1D, F). Cell migration was likewise attenuated upon FUT8 silencing. Transwell assays showed a significant reduction in the number of migrated cells, while wound-healing assays performed under serum-free conditions revealed delayed wound closure in FUT8-knockdown cells (Figs. 2E, G–I; S1E, G–I). Importantly, re-expression of FUT8^Res^ markedly restored migratory capacity in both assays, confirming that the observed effects were specifically attributable to FUT8 loss.

At the molecular level, Western blot analysis with densitometric quantification revealed that FUT8 knockdown increased the epithelial marker E-cadherin while reducing the mesenchymal markers N-cadherin and vimentin, consistent with suppression of epithelial–mesenchymal transition (EMT). These EMT-associated changes were partially reversed upon FUT8 rescue (Figs. 2J–K; S1J–K).

The in vitro findings were further validated in vivo. In a subcutaneous xenograft model, FUT8 knockdown significantly suppressed tumor growth compared with control cells (Fig. 2L, M). In addition, a tail-vein injection model using luciferase-labeled cells revealed a markedly reduced pulmonary metastatic burden in the FUT8-knockdown group, as assessed by bioluminescence imaging and histological analysis (Fig. 2N–Q).

Collectively, these results demonstrate that FUT8 depletion consistently attenuates ccRCC malignant phenotypes, including proliferation, migration, EMT-associated marker expression, tumor growth, and metastatic colonization. Importantly, the ability of FUT8^Res^ re-expression to rescue these phenotypes confirms the on-target specificity of FUT8 knockdown and supports a functional role for FUT8 in driving aggressive ccRCC progression.

### FUT8 drives HIF-1α-mediated metabolic reprogramming in ccRCC

**Fig. 3:**
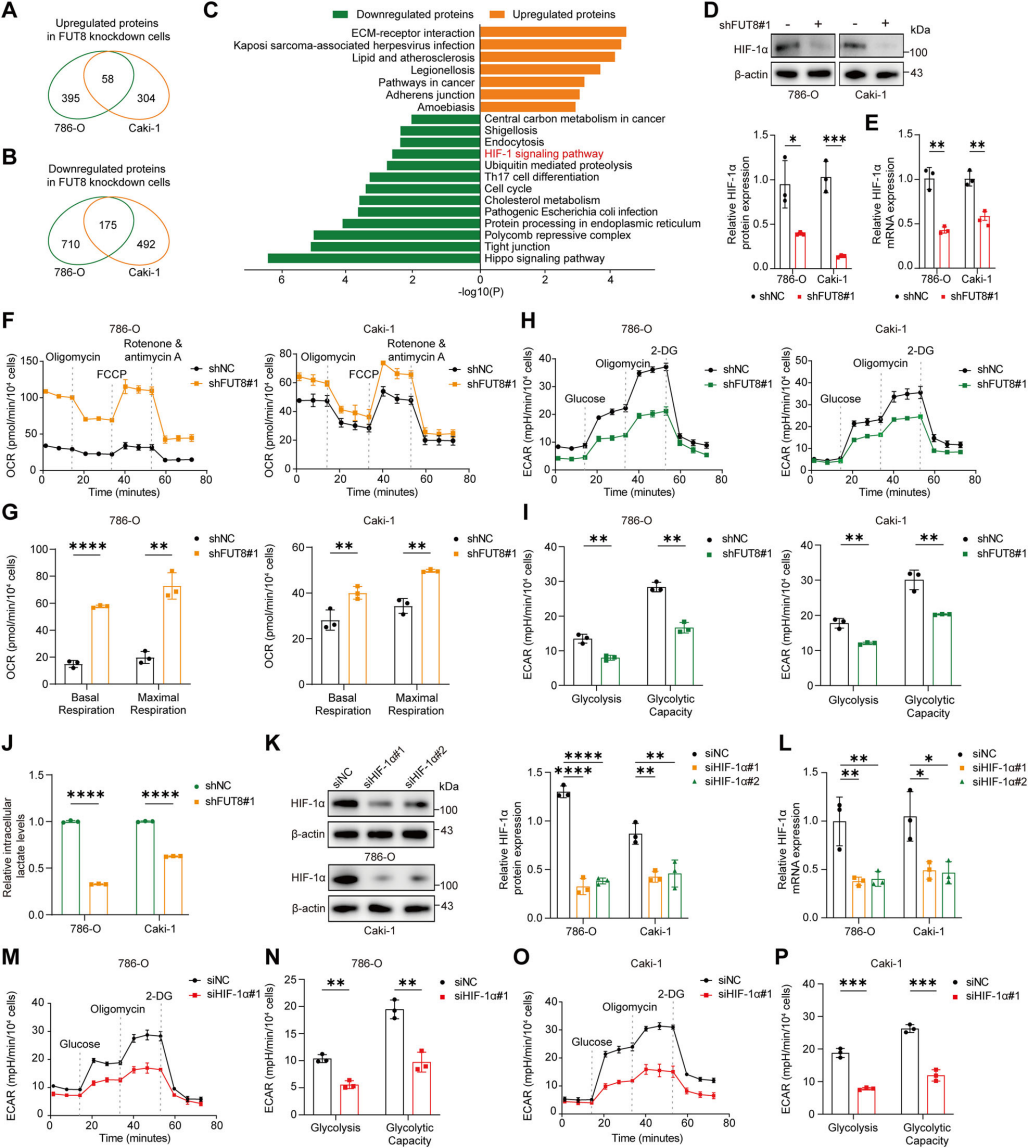
FUT8 drives HIF-1α-mediated metabolic reprogramming in ccRCC. **A**–**C** Quantitative proteomic analysis of FUT8-knockdown 786-O and Caki-1 cells. Venn diagrams show the overlap of upregulated **A** and downregulated **B** proteins identified in both cell lines. **C** KEGG pathway enrichment analysis of differentially expressed proteins, with upregulated and downregulated proteins analyzed separately. **D**, **E** HIF-1α protein and mRNA levels in control and FUT8-knockdown 786-O and Caki-1 cells measured by Western blotting and qRT-PCR. **F**–**I** Seahorse XF analysis of mitochondrial respiration and glycolysis in control and FUT8-knockdown 786-O and Caki-1 cells. Oxygen consumption rate (OCR) profiles and quantification of basal and maximal respiration are shown in (**F**, **G**). Extracellular acidification rate (ECAR) profiles and quantification of glycolysis and glycolytic capacity are shown in (**H**, **I**). **J** Intracellular lactate levels in control and FUT8-knockdown 786-O and Caki-1 cells. **K**, **L** Validation of HIF-1α knockdown efficiency by Western blotting and qRT-PCR in 786-O and Caki-1 cells transduced with two independent siRNAs targeting HIF-1α. **M**–**P** Seahorse XF ECAR analysis showing that silencing HIF-1α phenocopies FUT8 knockdown by suppressing glycolysis and glycolytic capacity in 786-O and Caki-1 cells. Data are presented as mean ± SD. Statistical significance was assessed using unpaired t tests or one-way ANOVA as appropriate. All in vitro experiments were performed in three independent biological replicates (*n* = 3). ns, *P* > 0.05; *P < 0.05; **P < 0.01; ***P < 0.001; ****P < 0.0001.

To elucidate the mechanism by which FUT8 promotes ccRCC malignancy, we performed quantitative proteomic profiling of 786-O and Caki-1 cells with or without FUT8 knockdown. Differentially expressed proteins shared between the two cell lines were identified and visualized using Venn diagrams, with upregulated and downregulated proteins analyzed separately (Fig. 3A, B). KEGG pathway enrichment analysis of the overlapping differentially expressed proteins revealed significant enrichment of hypoxia- and glycolysis-related pathways, including HIF-1 signaling (Fig. 3C), implicating coordinated regulation of glycolytic metabolism and hypoxic responses upon FUT8 depletion.

Consistent with this hypothesis, qRT-PCR confirmed reduced HIF-1α transcript levels, and immunoblotting verified concordant decreases in HIF-1α protein upon FUT8 silencing (Fig. 3D, E). Moreover, previous studies have suggested that YAP/TAZ, acting downstream of the Hippo pathway, can transcriptionally activate key glycolytic enzymes, thereby enhancing glucose uptake and lactate production [39–41], and thus may cooperate with HIF-1α signaling to promote metabolic reprogramming and ccRCC progression.

To functionally assess metabolic reprogramming, we measured oxygen-consumption rate (OCR) and extracellular acidification rate (ECAR) using a Seahorse XF96 analyzer. FUT8 knockdown increased OCR (Fig. 3F) and elevated both basal and maximal respiratory capacities (Fig. 3G), indicating enhanced oxidative phosphorylation. Conversely, ECAR was reduced (Fig. 3H) together with diminished glycolytic rate and capacity (Fig. 3I), a pattern consistent across both ccRCC cell lines. Together, these findings indicate that FUT8 supports a Warburg-like metabolic state characterized by elevated glycolysis and suppressed mitochondrial respiration, whereas FUT8 depletion shifts metabolism toward mitochondrial oxidation.

To assess the terminal product of glycolysis, intracellular lactate levels were quantified and found to be significantly reduced following FUT8 knockdown (Fig. 3J), consistent with the ECAR decrease. Enzymatic assays further showed that PK activity reduced upon FUT8 knockdown (Fig. S3A), whereas LDH activity remained unchanged (Fig. S3B). Elevated PK activity favors conversion of phosphoenolpyruvate to pyruvate; in ccRCC, high HIF-1α typically suppresses pyruvate dehydrogenase, restricting mitochondrial pyruvate entry. When LDH activity is unaltered, more cytosolic pyruvate would normally be reduced to lactate. Immunoblot analysis revealed no significant change in total PKM2 protein levels, while LDHA and LDHB expression showed only modest alterations (Fig. S3C), indicating that FUT8 primarily regulates glycolytic flux through functional modulation of key enzymes rather than changes in their abundance.

To determine whether HIF-1α mediates the metabolic effects downstream of FUT8, we silenced HIF-1α using two independent siRNAs. HIF-1α knockdown phenocopied FUT8 depletion by significantly suppressing ECAR, glycolytic rate, and glycolytic capacity in both 786-O and Caki-1 cells (Fig. 3M–P), supporting a critical role for HIF-1α in FUT8-driven metabolic reprogramming.

### Lactate mediates FUT8-dependent malignant phenotypes in ccRCC

**Fig. 4:**
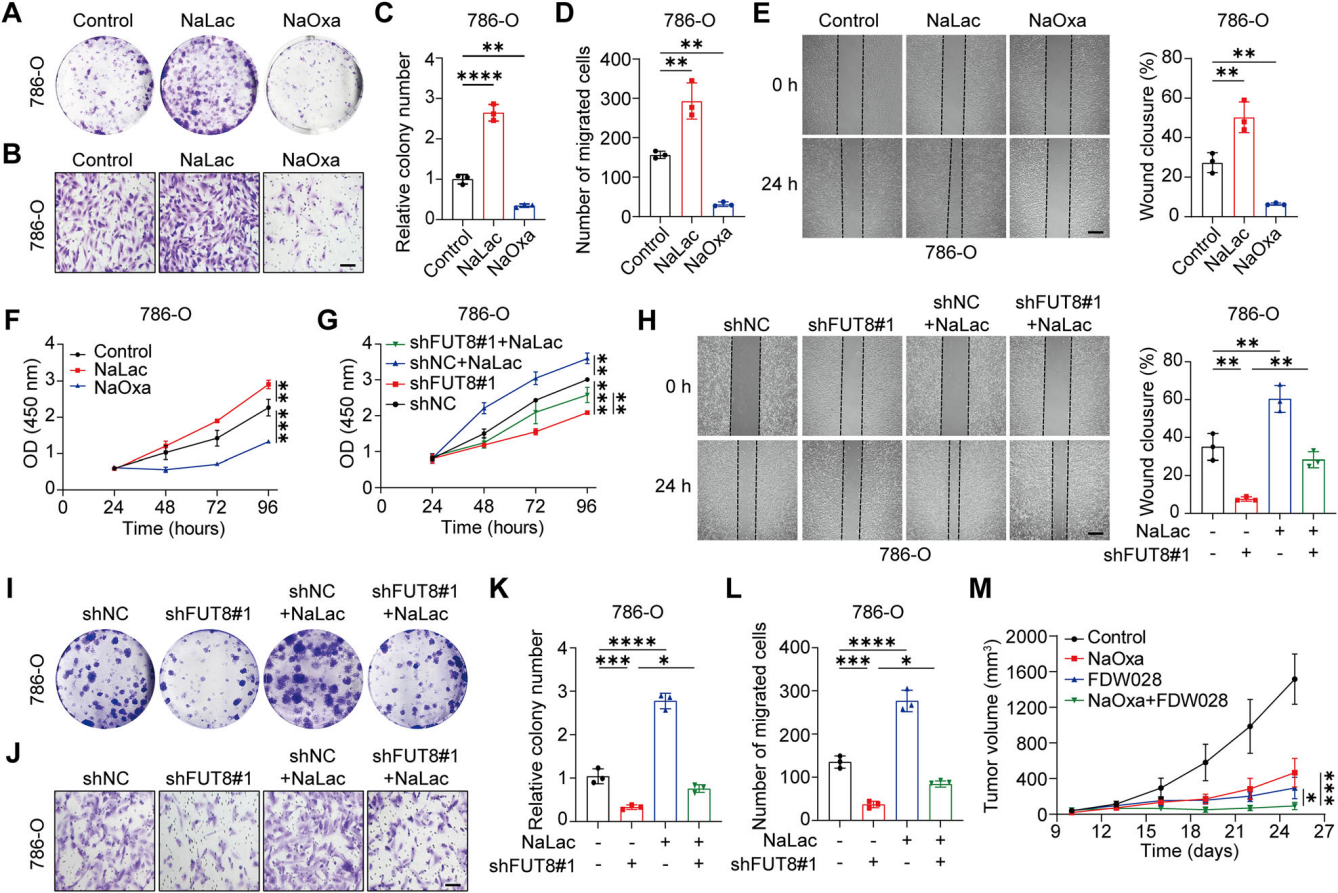
Lactate mediates FUT8-dependent malignant phenotypes in ccRCC. **A–F** Effects of lactate and lactate inhibition on ccRCC cell proliferation and migration. 786-O cells were treated with 20 mM sodium L-lactate (NaLac) or 10 mM sodium oxamate (NaOxa), and cell proliferation was assessed by colony formation assays (**A**, **C**) and CCK-8 assays (**F**). Cell migration was evaluated by Transwell assays (Scale bar: 50 µm) (**B**, **D**) and wound-healing assays (Scale bar: 50 µm) (**E**), with quantitative analyses shown. **G**–**L** Lactate partially rescues the impaired malignant phenotypes caused by FUT8 knockdown. 786-O cells expressing shNC or shFUT8#1 were treated with NaLac as indicated. Cell proliferation was measured by CCK-8 assays (**G**) and colony formation assays (**I**, **K**), while cell migration was assessed by wound-healing assays (**H**) and Transwell assays (**J**, **L**). **M** Combined inhibition of lactate metabolism and FUT8 suppresses tumor growth in vivo. Tumor growth curves of subcutaneous xenografts derived from 786-O cells treated with NaOxa, the FUT8 inhibitor FDW028, or the combination of NaOxa and FDW028 are shown (*n* = 5 per group). Data are presented as mean ± SD. Statistical significance was assessed using unpaired t tests or one-way ANOVA as appropriate. All in vitro experiments were performed in three independent biological replicates (*n* = 3). ns, *P* > 0.05; *P < 0.05; **P < 0.01; ***P < 0.001; ****P < 0.0001.

To assess the functional role of lactate in ccRCC progression, we pharmacologically modulated lactate levels in 786-O cells. Cells were treated with phosphate-buffered saline (PBS, control) 20 mM sodium L-lactate (NaLac), or 10 mM sodium oxamate (NaOxa, an LDHA inhibitor) for 24 h. Cell proliferation was assessed by CCK-8 and colony formation assays. NaLac treatment significantly enhanced proliferative capacity, whereas NaOxa markedly suppressed cell growth (Fig. 4A, C, F). Similarly, cell motility assays demonstrated that NaLac accelerated wound closure and promoted Transwell migration, whereas NaOxa inhibited both (Fig. 4B, D, E). Consistent results were also observed in Caki-1 cells (Fig. S4A–F). Collectively, these findings indicate that lactate actively promotes malignant behaviors in ccRCC, rather than serving merely as a metabolic by-product.

To directly test whether lactate acts downstream of FUT8, we performed lactate rescue experiments in FUT8-silenced cells. Compared with control cells, FUT8 knockdown significantly reduced proliferation and migration. Notably, exogenous lactate supplementation partially restored the impaired phenotypes caused by FUT8 depletion, as evidenced by increased CCK-8 signals, enhanced colony formation, and improved migratory capacity in both wound-healing and Transwell assays (Fig. 4G–L). The incomplete rescue is biologically consistent with FUT8 potentially contributing to ccRCC progression through both lactate-dependent and lactate-independent mechanisms. Collectively, these findings support lactate as a functional downstream mediator of FUT8-driven malignant behaviors.

Given the translational implications of targeting this axis, we next evaluated whether combined inhibition of lactate metabolism and FUT8 could suppress tumor growth in vivo. To evaluate its activity in ccRCC, we established subcutaneous 786-O xenografts in BALB/c nude mice and randomized them to PBS, NaOxa (500 mg/kg), FDW028 (10 mg/kg), or the combination (*n* = 5 per group), with longitudinal monitoring of tumor-volume (Fig. 4M). All interventions reduced tumor burden compared with PBS controls, with the combination of NaOxa and FDW028 producing the most pronounced suppression. Immunoblotting of harvested tumors revealed reduced pan-Kla across all treatment groups relative to controls (Fig. S4H), consistent with the observed tumor growth suppression. These findings indicate that simultaneously constraining lactate production (via NaOxa) and selectively inhibiting FUT8 (via FDW028) synergistically suppresses ccRCC growth by reducing global lactylation, thereby reinforcing the FUT8–lactate axis as a driver of ccRCC progression.

### FUT8 regulates pan-lysine lactylation and promotes PKM2 lactylation in ccRCC

**Fig. 5:**
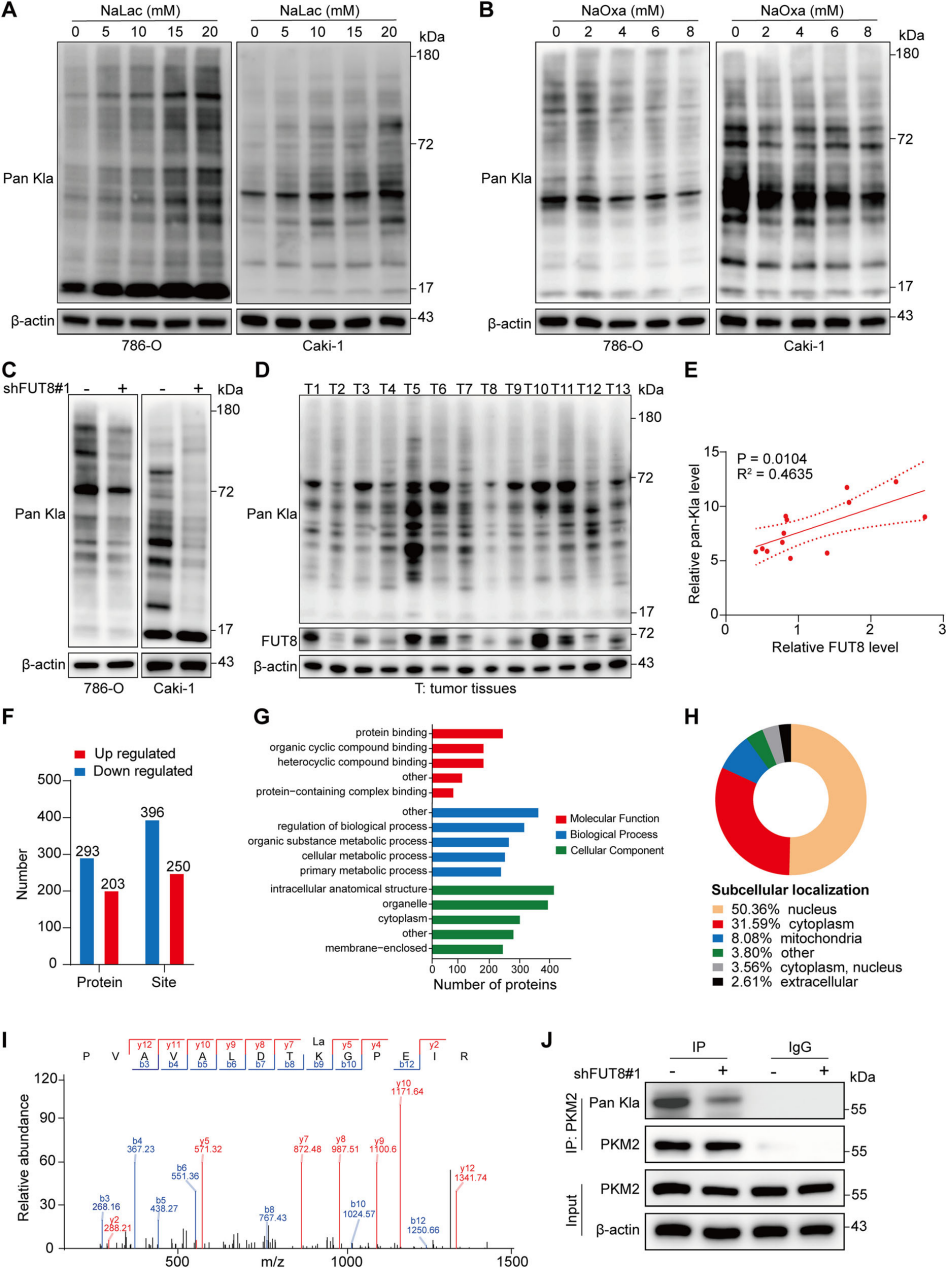
FUT8 regulates pan-lysine lactylation and promotes PKM2 lactylation in ccRCC. **A**, **B** pan-lysine lactylation **(**Pan-Kla) levels in 786-O and Caki-1 cells after 24 h exposure to graded concentrations of NaLac or NaOxa. **C** Western blot analysis of pan-Kla in FUT8-knockdown versus control 786-O and Caki-1 cells. **D**, **E** Analysis of pan-Kla levels and FUT8 expression in human ccRCC tumor tissues. Representative Western blots of pan-Kla and FUT8 in tumor samples (*n* = 13) (**D**), and correlation analysis between relative FUT8 and pan-Kla protein levels (**E**). **F**–**H** Lactyl-proteomic profiling of FUT8-regulated lactylation. Bar charts show the numbers of upregulated and downregulated lactylated proteins and modification sites (**F**). Gene Ontology (GO) enrichment analysis of FUT8-regulated lactylated proteins (**G**). Subcellular localization distribution of FUT8-regulated lactylated proteins (**H**). **I** Mass-spectrometric identification of PKM2 lysine 115 (K115) lactylation. **J** Immunoprecipitation of PKM2 followed by immunoblotting with anti-pan-Kla antibody in control and FUT8-knockdown 786-O cells, confirming reduced PKM2 lactylation upon FUT8 depletion.

To investigate the relationship between the FUT8, lactate, and Kla in ccRCC, we treated 786-O and Caki-1 cells with increasing concentrations of NaLac or NaOxa for 24 h. Immunoblotting analysis revealed a dose-dependent increase in pan-Kla with increasing NaLac, and a corresponding decrease with NaOxa (Fig. 5A, B), indicating a positive association between intracellular lactate and pan-Kla. Additionally, FUT8 knockdown markedly reduced pan-Kla in both cell lines, whereas control cells maintained higher baseline lactylation (Fig. 5C). Combined with our prior evidence that FUT8 enhances glycolytic flux and lactate accumulation, these data support a FUT8–lactate–lactylation regulatory axis. In human ccRCC tumor samples (*n* = 13), FUT8 protein expression positively correlated with pan-Kla levels, with higher FUT8 abundance associated with increased pan-Kla (Fig. 5D, E). In addition, suppression of HIF-1α led to a clear reduction in pan-Kla levels (Fig. S3D), consistent with reduced FUT8-driven lactylation. Together with our previous findings that FUT8 promotes glycolysis and lactate production, these data support a regulatory link between FUT8 activity, lactate accumulation, and protein lactylation in ccRCC.

To systematically characterize FUT8-regulated lactylation events, we performed lactyl-proteomic profiling in control and FUT8-knockdown cells. Quantitative analysis identified a substantial number of lactylated proteins and modification sites that were significantly altered upon FUT8 depletion (Fig. 5F). Gene Ontology enrichment analysis revealed that FUT8-regulated lactylated proteins were enriched in metabolic processes and intracellular compartments relevant to glycolytic regulation (Fig. 5G, H).

Among these candidates, PKM2, a key glycolytic enzyme, was identified as a prominent lactylated protein. Tandem mass spectrometry confirmed lactylation of PKM2 at lysine 115 (K115) (Fig. 5I). Immunoprecipitation of PKM2 followed by pan-Kla immunoblotting further demonstrated that PKM2 lactylation was markedly reduced upon FUT8 knockdown (Fig. 5J), indicating that FUT8 promotes PKM2 lactylation in ccRCC cells.

### PKM2 K115 lactylation promotes glycolysis and malignant phenotypes in ccRCC

**Fig. 6:**
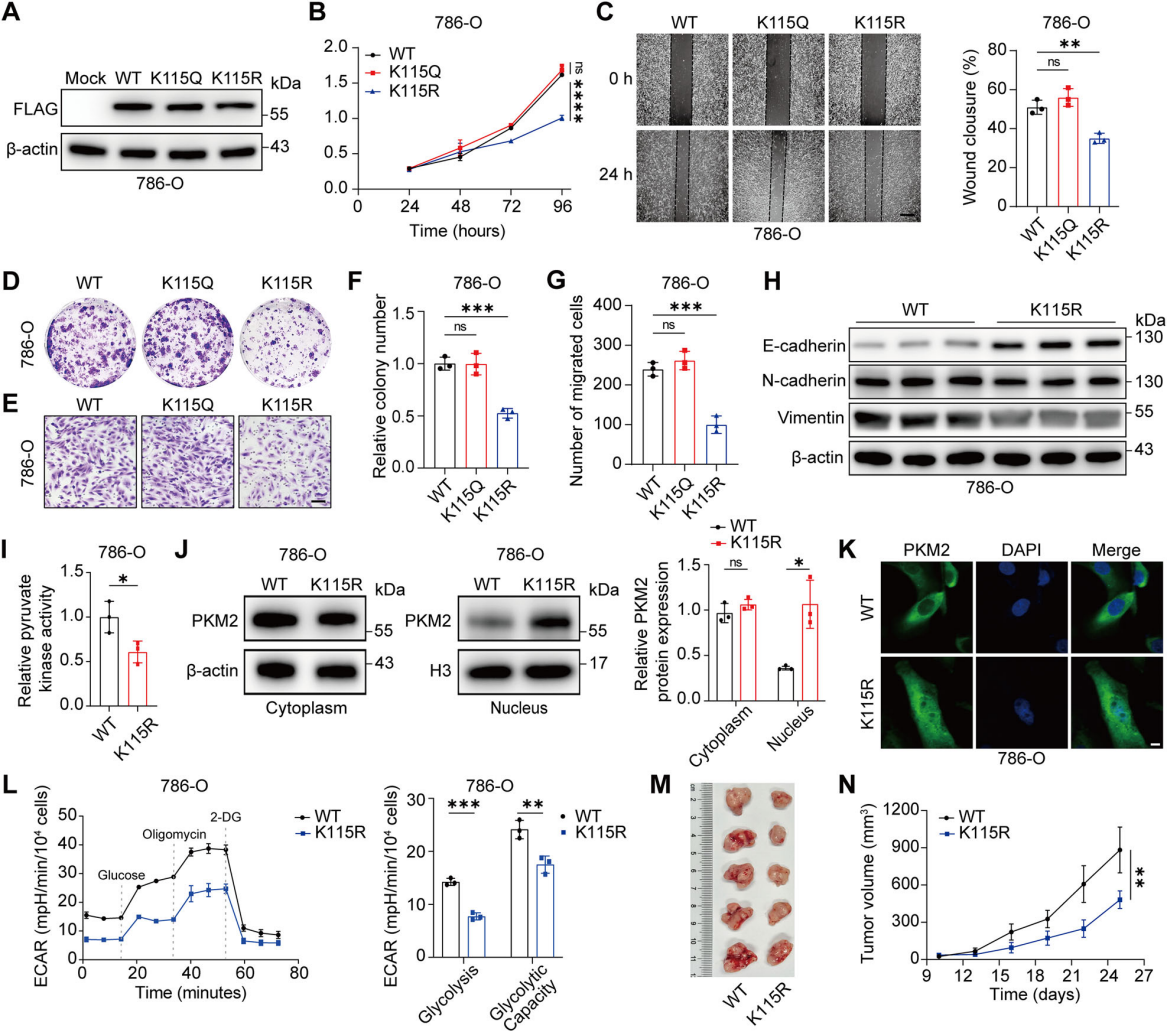
PKM2 K115 lactylation promotes glycolysis and malignant phenotypes in ccRCC. **A**–**G** Functional characterization of PKM2 lactylation at K115. FLAG-tagged PKM2 wild-type (WT), lactylation-mimetic mutant (K115Q), and lactylation-deficient mutant (K115R) were expressed in 786-O cells. Comparable expression levels were confirmed by Western blotting (**A**). Cell proliferation was assessed by CCK-8 assays (**B**) and colony formation assays (**D**, **F**). Cell migration was evaluated by wound-healing assays (Scale bar: 50 µm) (**C**) and Transwell assays (Scale bar: 50 µm) (**E**, **G**). Quantitative analyses are shown for each assay. **H** Western blot analysis of EMT markers, including E-cadherin, N-cadherin, and vimentin, in 786-O cells expressing PKM2 WT or K115R. **I** Pyruvate kinase enzymatic activity in 786-O cells expressing PKM2 WT or K115R. **J**–**K** Subcellular localization of PKM2 in 786-O cells expressing PKM2 WT or K115R. Cytoplasmic and nuclear fractions were analyzed by Western blotting using β-actin and histone H3 as cytoplasmic and nuclear markers, respectively (**J**). Representative immunofluorescence images showing PKM2 localization are shown in (**K**) (scale bar: 10 µm). **L** Seahorse XF analysis of ECAR profiles and quantification of glycolysis and glycolytic capacity in 786-O cells expressing PKM2 WT or K115R. **(M–N)** In vivo tumorigenic potential of PKM2 WT- and K115R-expressing cells. Representative images of subcutaneous xenograft tumors (**M**) and corresponding tumor growth curves (**N**) from BALB/c nude mice injected with the indicated cells (*n* = 5 per group). Data are presented as mean ± SD. Statistical significance was assessed using unpaired t tests or one-way ANOVA as appropriate. All in vitro experiments were performed in three independent biological replicates (*n* = 3). ns, *P* > 0.05; *P < 0.05; **P < 0.01; ***P < 0.001; ****P < 0.0001.

To determine whether PKM2 K115 lactylation functions as a downstream effector of FUT8-driven oncogenic signaling, we generated FLAG-tagged PKM2 wild-type (WT), lactylation-deficient (K115R), and lactylation-mimetic (K115Q) constructs and established stable overexpression models in 786-O cells. Western blot analysis confirmed comparable expression levels among the three PKM2 variants, enabling direct functional comparisons (Fig. 6A). Given the HIF-high context characteristic of ccRCC, we anticipated that K115Q would function as a lactylation-mimetic state comparable to WT, whereas K115R would model a de-lactylated lysine with tumor-suppressive potential.

Functional assays revealed that K115R markedly impaired proliferation compared with WT, whereas K115Q exhibited no difference relative to WT (Fig. 6B). Consistently, colony formation assays demonstrated reduced colony number and size in K115R-expressing cells, but not in K115Q-expressing cells (Fig. 6D, F). Cell motility assays yielded concordant results: K115R reduced wound closure and Transwell migration, while K115Q mirrored WT (Fig. 6C, E, G).

At the molecular level, PKM2 K115R expression was associated with an EMT-suppressive pattern, characterized by increased E-cadherin and decreased N-cadherin and vimentin compared with WT (Fig. 6H). Because PKM2 is a key glycolytic enzyme, we next measured PK activity and found that PKM2 K115R significantly reduced PK enzymatic activity relative to WT (Fig. 6I). Moreover, subcellular fractionation demonstrated an increased nuclear fraction of PKM2 in K115R-expressing cells, and immunofluorescence staining visually confirmed enhanced nuclear localization of PKM2 K115R compared with WT (Fig. 6J, K). To directly evaluate the impact of K115 lactylation on glycolytic flux, we performed Seahorse ECAR analysis. Compared with WT, PKM2 K115R expression significantly reduced ECAR, including basal glycolysis and glycolytic capacity (Fig. 6L), indicating impaired glycolytic activity at the cellular level.

Finally, to establish the physiological relevance of PKM2 K115 lactylation, we performed a subcutaneous xenograft assay. 786-O cells expressing PKM2 K115R formed significantly smaller tumors and exhibited reduced tumor growth compared with WT controls (Fig. 6M, N), demonstrating that loss of PKM2 K115 lactylation suppresses tumorigenesis in vivo. Collectively, these results indicate that PKM2 K115 lactylation supports glycolytic activity and malignant phenotypes in ccRCC.

## Discussion

FUT8 has been widely implicated in cancer progression through its canonical role in protein core fucosylation, enhancing signaling of EGFR, TGF-β1, and integrins [32, 42, 43]. However, its contribution to ccRCC has remained largely unexplored. Given that VHL loss constitutively stabilizes HIF proteins in ccRCC, where HIF-2α is generally dominant but HIF-1α remains a key regulator of glycolysis, we focused on HIF-1α–dependent metabolic changes [44–46]. We find that FUT8 expression correlates with enrichment of HIF-1α-associated pathways, increased glycolytic flux, and elevated lactate burden. These data suggest that FUT8 amplifies oncogenic signaling not only at the receptor level but also through downstream metabolic consequences.

Here, we demonstrate that FUT8 amplifies the hypoxia–glycolysis axis in the VHL-deficient context, driving lactate accumulation and global protein lactylation. Notably, lactylation of PKM2 at K115 emerges as a central functional node that links altered metabolism to EMT. Mechanistically, K115 lactylation is associated with reduced pyruvate kinase activity, increased nuclear localization of PKM2, and the induction of EMT-related transcriptional programs. Conversely, decreased lactate levels attenuate K115 lactylation and the transcriptional activity of EMT-inducing factors such as Snail1 and Twist1 [47, 48], ultimately constraining EMT and tumor progression. These findings highlight lactate not merely as a metabolic by-product but as a signaling intermediate that shapes cellular phenotype via non-histone protein lactylation. Alanyl-tRNA synthetase 1 (AARS1) functions as a bona fide lactyltransferase, employing an ATP-dependent two-step mechanism wherein lactate and ATP first generate a lactyl-AMP intermediate, which then donates the lactyl group to target proteins [49]. Structurally, the catalytic domain of FUT8 contains a Rossmann-like fold that accommodates GDP-fucose and is inherently suited to bind adenine nucleotide–containing molecules such as ATP, ADP, AMP, and CoA [50, 51]. By analogy to AARS1, we therefore propose that FUT8 may act not only as a lactyltransferase but also as a sensor of intracellular lactate.

Previous reports have described PKM2 lactylation at distinct residues, such as K62 in macrophages and K433 in bladder cancer, each exerting context-dependent effects on enzymatic activity and nuclear translocation. In contrast, our study identifies K115 lactylation in ccRCC, highlighting a novel site-specific mechanism by which FUT8-driven lactate reprogramming promotes malignant progression. This site-specific modification expands the repertoire of PKM2 regulation and underscores the diversity of lactylation-mediated signaling across tissues and tumor types.

These findings also provide a rationale for the clinical heterogeneity observed in ccRCC: despite the high prevalence of VHL loss, patient outcomes vary considerably, and FUT8 expression may represent one determinant of this variability. Accordingly, FUT8 abundance and its associated lactylation signatures may serve as biomarkers for patient stratification and as therapeutic vulnerabilities in the hypoxic ccRCC microenvironment.

Together, our work delineates a FUT8–HIF-1α–lactate–PKM2 axis that integrates metabolic reprogramming with PTM to drive EMT and invasion in ccRCC. By extending the oncogenic role of FUT8 from receptor signaling to metabolic–PTM regulation, this study highlights lactylation, particularly PKM2 K115 modification, as a mechanistic bridge linking glycolytic stress to tumor-promoting phenotypes.

## Materials and methods

### Patients and specimens

Clinical tissue specimens (30 ccRCC tumors and matched adjacent normal tissues) were obtained from the Department of Urology, Shenzhen Second People’s Hospital. Detailed clinicopathological characteristics of the patients are summarized in Table S1 (Supplementary material). Written informed consent was obtained from all participating patients prior to tissue collection. The study protocol was approved by the Ethics Committee of the First Affiliated Hospital of Shenzhen University (No. 20211011009).

### Cell culture and treatment

All cell lines were obtained from the American Type Culture Collection (ATCC, Manassas, VA, USA). 786-O cells were maintained in RPMI-1640 medium (Gibco, USA), and Caki-1 cells were cultured in McCoy’s 5 A medium (Gibco, USA). Both culture media were supplemented with 10% heat-inactivated fetal bovine serum (FBS; Gibco), 100 U/mL penicillin, and 100 μg/mL streptomycin (Gibco). Cells were incubated in an incubator (37 °C, 5% CO_2_). To assess the role of lactate in ccRCC progression, intracellular lactate levels were pharmacologically manipulated in 786-O and Caki-1 cells. Cells were treated for 24 h with either phosphate-buffered saline (PBS; Servicebio) as control, 20 mM sodium L-lactate (L7022-5G; Sigma-Aldrich), or 10 mM sodium oxamate (HY-W013032A; MedChemExpress).

### Animal model and treatment

All animal experiments were conducted in accordance with institutional guidelines and the rules of the Animal Ethical and Welfare Committee in the China Technology Industry Holdings (Shenzhen) Co., Ltd. (project Authorization No. 20250062). Male BALB/c nude mice (4–6 weeks) were purchased from GemPharmatech (Guangdong, China). FUT8-knockdown or control 786-O cells were resuspended in 100 μL PBS and subcutaneously injected into the right side of BALB/c nude mice (3 × 10^6^ cells per mouse, *n* = 5 per group).

For the pulmonary metastasis model, 3 × 10^6^ FUT8-knockdown or control 786-O cells in 100 μL PBS were injected into the tail vein of a separate cohort of BALB/c nude mice (*n* = 5 per group). After 6 weeks, lungs were collected, sectioned into three parts, and subjected to hematoxylin and eosin (H&E) staining to evaluate metastatic tumor nodules.

In the therapeutic intervention study, intraperitoneally (i.p.) drug administration was initiated ~10 days after cell implantation, when tumors reached ~5 mm in diameter. Mice received sodium oxamate (500 mg/kg) or FDW028 (10 mg/kg; Selleck), dissolved in the manufacturer-recommended vehicle every other day. Tumor volumes were measured every three days using a digital caliper and calculated using the formula: Volume = 0.5 × length (mm) × [width (mm)]².

### Western blotting

Cells were lysed in RIPA buffer (Yeasen, 20101ES60) supplemented with PMSF (Yeasen, 58076ES60) to extract total proteins. For tumor tissues, samples were minced, rinsed with cold PBS, and homogenized on ice using a tissue homogenizer until no obvious solid remained. The homogenates were incubated on ice for 5 min, and the supernatant was carefully transferred to pre-chilled centrifuge tubes. Samples were centrifuged at 500 × *g* for 2–3 min at 4 °C, and the pellets were resuspended in RIPA buffer. Protein lysates were mixed with loading buffer (Yeasen, 20315ES05), boiled to denature proteins, separated by SDS-PAGE, and transferred onto polyvinylidene fluoride (PVDF) membranes. The membranes were blocked with 5% non-fat milk for 1 h at room temperature and incubated overnight at 4 °C with the indicated primary antibodies. After washing with Tris-buffered saline containing 0.1% Tween 20 (TBST), membranes were incubated with HRP-conjugated secondary antibodies, washed again with TBST, and developed using an enhanced chemiluminescence substrate. Signals were detected using a chemiluminescence imaging system (Amersham Imager 680, USA). Primary antibodies used in this study included anti-FUT8 (1:1000, Proteintech, 66118-1-Ig), anti-E-cadherin (1:10000, Proteintech, 20874-1-AP), anti-N-cadherin (1:5000, Proteintech, 22018-1-AP), anti-Vimentin (1:1000, ABclonal, A2584), anti- HIF-1 alpha (1:5000, Proteintech, 20960-1-AP), anti-PKM2 (1:1000, Selleck, F23E18), anti-LDHA (1:1000, Cell Signaling Technology, 3582), anti-LDHB (1:1000, PTM Bio, PTM-5869), pan anti-Kla (1:1000, PTM Bio, PTM-1401RM), anti-FLAG (1:10000, Proteintech, 66008-4-Ig), and anti-beta Actin (1:2000, Servicebio, ZB15001-HRP-100). HRP-conjugated secondary antibodies were used at 1:2000 (ABclonal, AS014, AS003).

### Immunohistochemistry

Formalin-fixed, paraffin-embedded tissues were sectioned, deparaffinized, and subjected to antigen retrieval. Sections were incubated overnight at 4 °C with primary antibody against FUT8 (1:1000, Proteintech, 66118-1-Ig), followed by incubation with HRP-conjugated secondary antibodies (1:2000, ABclonal, AS014) for 1 h at room temperature. Signals were developed with DAB, counterstained with hematoxylin, dehydrated through graded alcohols, cleared in xylene, and mounted for microscopic analysis.

### Cell transfection and lentivirus infection

For FUT8 knockdown, short hairpin RNA (shRNA) lentiviral constructs targeting human FUT8 (shFUT8#1 and shFUT8#2) and a non-targeting control shRNA (shNC) were designed and generated by GeneChem (Shanghai, China) and cloned into the pLKO.1-puro-GFP vector. Stable cell lines were selected by treatment with puromycin (2 μg/mL) for 10 days. For rescue experiments, shFUT8#1 cells were transduced with an shRNA-resistant FUT8 expression construct (FUT8^Res^), in which silent mutations were introduced into the shRNA target site without altering the amino acid sequence. Cells transduced with the corresponding empty vector served as controls. For transient silencing of HIF-1α, cells were transfected with small interfering RNAs (siRNAs) targeting HIF-1α or a scrambled negative control siRNA (Ribobio, Guangzhou, China) using Lipofectamine RNAiMAX (Invitrogen, USA). PKM2 WT and mutant (FLAG-tagged) expression plasmids were synthesized by BGI Genomics (Beijing, China) and transfected into 786-O cells. Lentiviral constructs encoding wild-type FUT8 or FUT8^Res^ were generated by OBiO Technology (Shanghai, China). All the shRNA and siRNA sequences are provided in the Table S2.

### Quantitative real-time PCR (qRT-PCR)

Total RNA was extracted from pretreated 786-O and Caki-1 cells using the MolPure^®^ Flash Cell/Tissue Total RNA Kit (Yeasen, 19221ES50). Complementary DNA (cDNA) was synthesized using the Hifair^®^ III 1st Strand cDNA Synthesis SuperMix for qPCR (Yeasen, 11141ES10). The qRT-PCR was performed with Hieff UNICON^®^ Universal Blue qPCR SYBR Green Master Mix (Yeasen, 11184ES08) on a real-time PCR system (QuantStudio 3, Applied Biosystems, USA). Thermocycling conditions were as follows: initial denaturation at 95 °C for 2 min, followed by 35 cycles of 95 °C for 10 s and 60 °C for 30 s. Relative gene expression was normalized to β-Actin. Primers sequences are listed in Table S2.

### Cell counting kit-8 (CCK-8) assay

Cells were seeded into 96-well plates and treated according to the experimental protocol. Cell viability was measured using the CCK-8 kit (Techisunbio, TQ-BG-025). Absorbance at 450 nm (OD450) was recorded.

### Clonal formation assay

Cells were seeded in 6-well plates at a density of 1000 cells per well and incubated under the indicated experimental conditions for ~14 days. Colonies were fixed with 4% paraformaldehyde, stained with crystal violet solution, and visualized. Colony areas were quantified using Image J software.

### Wound healing assay

Cells were seeded in 6-well plate and grown to a confluent monolayer. A linear scratch was generated with a 200 μL sterile pipette tip, after which the wells were gently washed with PBS to remove detached cells. The medium was then replaced with serum-free culture medium to minimize the influence of cell proliferation on wound closure. Images of the same wound area were captured at 0 h and 24 h using a phase-contrast microscope. Wound closure was quantified using Image J software.

### Transwell assay

Cell migration was assessed using 24-well cell culture chambers (Corning, USA). A total of 5 × 10^4^ cells suspended in 200 μL of FBS-free medium were added to the upper chamber, while the lower chamber was filled with 600 μL of medium containing 10% FBS. After 48 h of incubation, cells that migrated to the lower surface of the chamber were fixed with 4% paraformaldehyde and stained with crystal violet. Stained cells were photographed under a microscope, and the number of migrated cells was quantified using Image J software.

### LC-MS/MS analysis

4D label-free quantitative LC-MS/MS analysis was conducted by PTM Bio (Hangzhou, China). To enrich modified peptides, tryptic peptides dissolved in NETN buffer (100 mM NaCl, 1 mM EDTA, 50 mM Tris-HCl, 0.5% NP-40, pH 8.0) were incubated with pre-washed antibody beads (PTM-1401RM, PTM Bio) at 4 °C overnight with gentle shaking. Then the beads were washed for four times with NETN buffer and twice with H_2_O. The bound peptides were eluted from the beads with 0.1% trifluoroacetic acid. Finally, the eluted fractions were combined and vacuum-dried. For LC-MS/MS analysis, the resulting peptides were desalted with C18 ZipTips (Millipore) according to the manufacturer’s instructions. The peptides were subjected to capillary source followed by the timsTOF Pro (Bruker Daltonics) mass spectrometry.

### Seahorse XF ECAR and OCR measurement

The extracellular acidification rate (ECAR) and oxygen-consumption rates (OCR) were measured using an XF96 Extracellular Flux analyzer (Agilent Technologies) with the Agilent Seahorse XF Cell Mito Stress Test and XF Glycolysis Stress Test kit. Cells were cultured in indicated medium for at least 48 h before the assay. A total of 2 × 10^4^ cells in 200 μL of the same medium were seeded into XF96 cell culture microplate 16 h before measurement. On the day following cell seeding, the culture medium was replaced with phenol red-free Seahorse XF DMEM medium (pH 7.4) supplemented with 2 mM glutamine, and cells were equilibrated for 1 h at 37 °C in a non- CO_2_ incubator. Subsequent measurements were performed according to the manufacturer’s instructions.

### Quantification of lactate

Intracellular lactate levels were measured using a colorimetric lactate assay according to the manufacturer’s instructions (CheKine™ Micro Lactate Assay Kit, KTB1100).

### Pyruvate kinase (PK) activity assay

Cells were lysed, and the supernatant was used to measure PK activity following the manufacturer’s protocol (CheKine™ Micro Pyruvate Kinase Assay Kit, KTB1120). Enzymatic activity was normalized to total protein concentration.

### Lactate dehydrogenase (LDH) activity assay

Cell lysates were centrifuged to obtain the supernatant, which was subjected to LDH activity measurement using the CheKine™ Micro Lactate Dehydrogenase Assay Kit (KTB1110) following the manufacturer’s protocol. Reaction mixtures were incubated with the samples, and absorbance was measured at 450 nm. Protein concentrations were equalized across samples prior to analysis.

### Immunoprecipitation (IP)

IP was performed using the Protein A/G Immunoprecipitation Kit (Yeasen, 36421ES40). Cells were lysed in RIPA buffer containing protease inhibitors on ice for 10 min, and the supernatant was collected by centrifugation. Supernatants were incubated with the indicated primary antibodies at room temperature for 10 min, followed by incubation with Protein A/G for 2 h. The immune complexes were collected, washed repeatedly with TBS, and eluted by boiling in SDS-PAGE loading buffer.

### Nuclear and cytoplasmic protein extraction

For subcellular fractionation, nuclear and cytoplasmic proteins were isolated using the Nuclear and Cytoplasmic Protein Extraction Kit (Beyotime, P0028) according to the manufacturer’s instructions.

### Immunofluorescence

Cells were seeded onto sterile glass coverslips placed in 24-well plates and cultured until 60–70% confluence. After the indicated treatments or genetic manipulations, cells were washed twice with PBS and fixed with 4% paraformaldehyde for 15 min at room temperature. Following fixation, cells were washed three times with PBS and permeabilized with 0.25% Triton X-100 in PBS for 10 min. Non-specific binding was blocked by incubating the cells with 3% bovine serum albumin in PBS for 1 h at room temperature. Cells were then incubated overnight at 4 °C with the indicated primary antibodies diluted in blocking buffer. After washing three times with PBS, cells were incubated with appropriate fluorophore-conjugated secondary antibodies for 1 h at room temperature in the dark. Nuclei were counterstained with DAPI for 10 min at room temperature in the dark, followed by three washes with PBS. Coverslips were mounted onto glass slides using anti-fade mounting medium and sealed. Fluorescence images were acquired using a confocal laser scanning microscope (Zeiss LSM series) under identical acquisition settings for all experimental groups.

### Survival analysis

Patient survival analyses and Kaplan–Meier curves for the indicated ccRCC cohorts (KIRC) were generated using TCGA data (Table S4). P-values were calculated using the log-rank test. Differential mRNA expression of FUT8 between tumor and normal kidney tissues (Table S3) was obtained from TCGA and GTEx datasets. All data processing and visualization were performed in R version 4.4.3.

### Statistical analysis

Graphs and statistical analyses were performed using GraphPad Prism 9.0 software. Data are presented as mean ± SD. Comparisons between two groups were conducted using an unpaired Student’s t-test, whereas multiple-group comparisons were analyzed using one-way analysis of variance (ANOVA). A *P* value of < 0.05 was considered statistically significant.

## Supplementary information

Supplementary information

Table S1

Table S2

Table S3

Table S4

Original western blots

## Data Availability

The datasets used and analyzed during the current study are available from the corresponding author on reasonable request.
