# Supplementary material for: FUT8 reprograms glycolytic metabolism to promote PKM2 lactylation and drive clear cell renal cell carcinoma progression
Source: Cell Death Discov. 2026 Mar 19;12:146. doi: 10.1038/s41420-026-03013-1 (PMC13039778; doi:10.1038/s41420-026-03013-1)
Supplement: Supplementary file 1 — Supplementary information [file 41420_2026_3013_MOESM1_ESM.docx]

**Supplementary information**

**FUT8 reprograms glycolytic metabolism to promote PKM2 lactylation and drive clear cell renal cell carcinoma progression**

Zikai Guo ^a,b,1^, Hongxiao Jiang ^b,f,1^, Xu Wang ^g,1^, Ke Xuan ^b^, Huidong Zhong ^h^, Chengxi Liu ^i^, Mengkai Zhang ^b^, Zhichao Li ^b,*^, Weiren Huang ^a,b,c,d,e,*^, Yangyang Sun ^c,*^

^a^ Department of Urology, the First Affiliated Hospital of Anhui Medical University, Hefei, China

^b^ The First Affiliated Hospital, Shenzhen University; Shenzhen Second People's Hospital; Medical Innovation Technology Transformation Center of Shenzhen Second People's Hospital, Shenzhen, China.

^c^ State Key Laboratory of Quantitative Synthetic Biology, Shenzhen Institute of Synthetic Biology, Shenzhen Institutes of Advanced Technology, Chinese Academy of Sciences, Shenzhen, China.

^d^ Guangdong Key Laboratory of Systems Biology and Synthetic Biology for Urogenital Tumors, Shenzhen, China.

^e^ GuangDong Engineering Technology Research Center for clinical application of cancer genome, Shenzhen, China.

^f^ Guangxi University of Chinese Medicine, Nanning, China.

^g^ The Third Affiliated Hospital of Anhui Medical University (The First People’s Hospital of Hefei), Hefei, China.

^h^ Department of Oncology, Ji'an Central Hospital, Ji'an, China.

^i^ Department of Applied Biology and Chemical Technology, Food Safety and Technology Research Centre, and Research Centre for Chinese Medicine Innovation, The Hong Kong Polytechnic University, Hung Hom, Kowloon, Hong Kong SAR, China.

^1^ These authors contributed equally to this work.


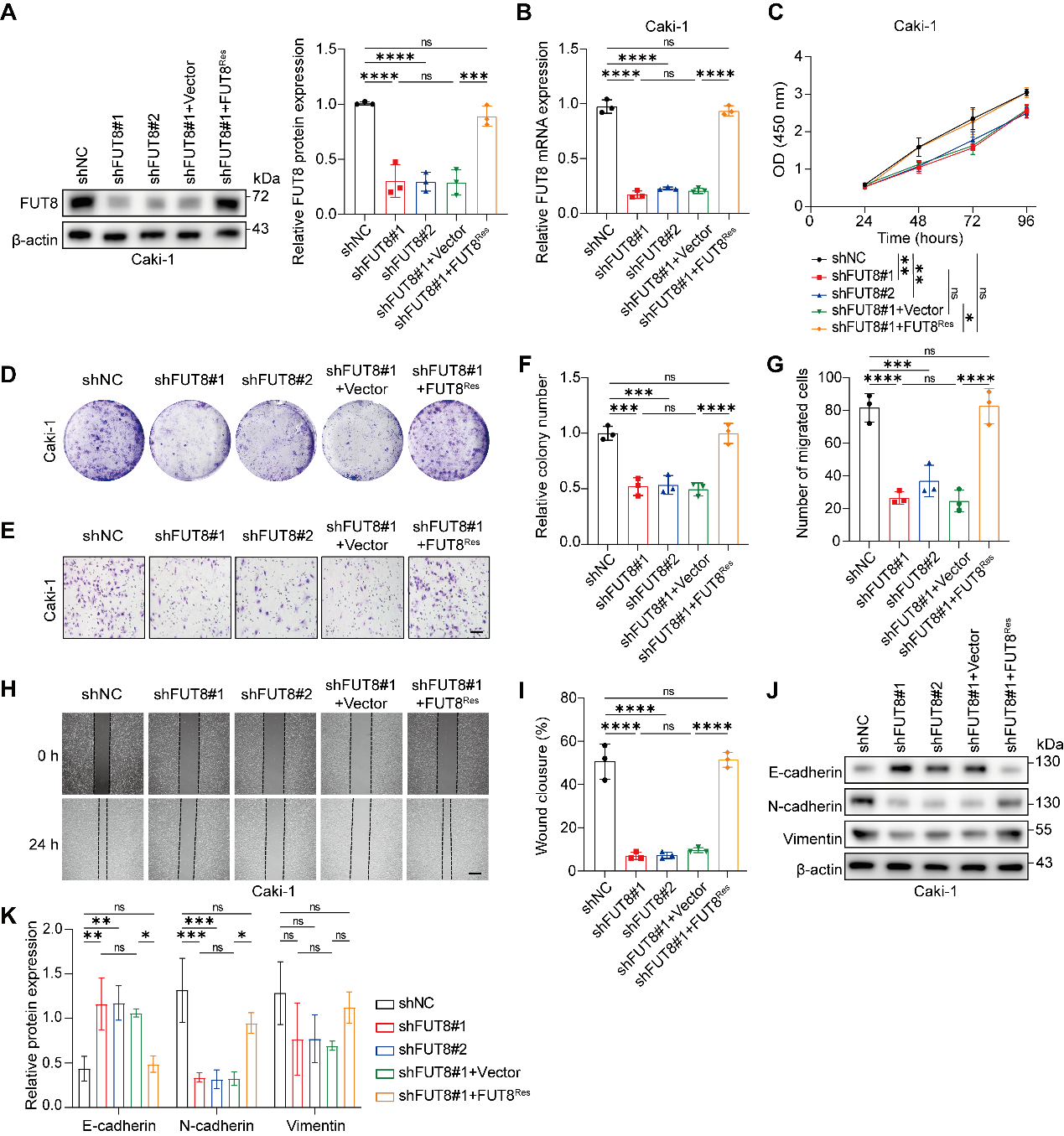


**Fig. S1 Knockdown of FUT8 suppresses Caki-1 cells proliferation and migration.** **(A–B)** Verification of FUT8 knockdown and rescue efficiency in Caki-1 cells by Western blotting and qRT-PCR. **(C–D, F)** Cell proliferation of Caki-1 cells under the indicated conditions was assessed by CCK-8 and colony formation assays, with quantitative analysis of colony numbers. **(E, G–I)** Cell migration of Caki-1 cells under the indicated conditions was evaluated by Transwell assays (scale bar: 50 µm) and wound-healing assays (scale bar: 50 µm), with quantification of migrated cells and wound closure. **(J–K)** Western blot analysis and densitometric quantification of epithelial–mesenchymal transition (EMT) markers, including E-cadherin, N-cadherin, and vimentin, in control, FUT8-knockdown, and FUT8-rescued Caki-1 cells. Data are presented as mean ± SD. Statistical significance was assessed using unpaired t tests or one-way ANOVA as appropriate. All in vitro experiments were performed in three independent biological replicates (n = 3). ns, P > 0.05; *P < 0.05; **P < 0.01; ***P < 0.001; ****P < 0.0001.


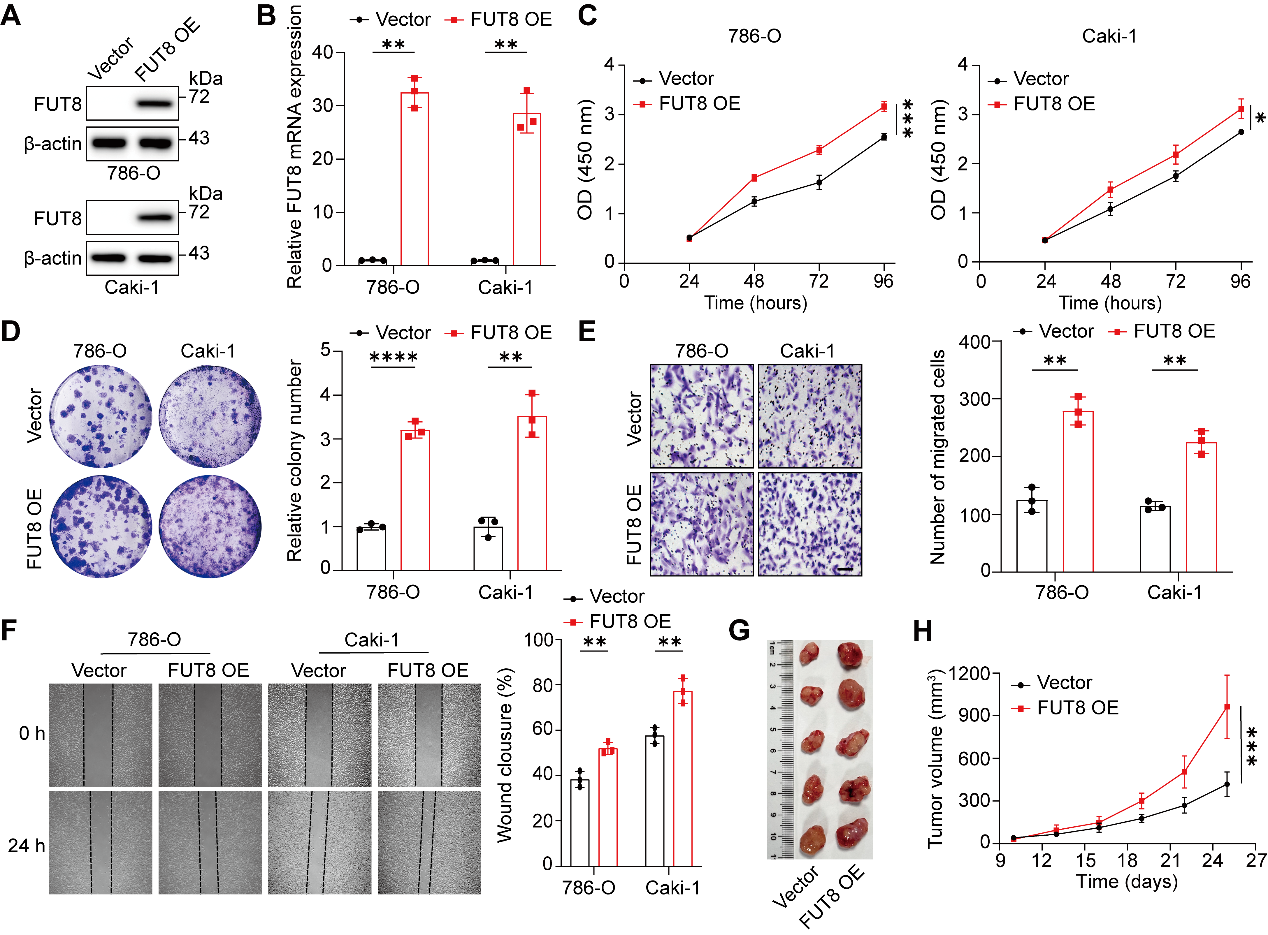


**Fig. S2 Overexpression of FUT8 promotes malignant phenotypes in ccRCC. (A–B)** Verification of FUT8 overexpression in 786-O and Caki-1 cells by Western blotting **(A)** and qRT-PCR **(B)**. Cells were transduced with empty vector (Vector) or FUT8 overexpression construct (FUT8 OE). **(C–D)** Cell proliferation of control and FUT8-overexpressing 786-O and Caki-1 cells assessed by CCK-8 assays **(C)** and colony formation assays **(D)**, with quantitative analysis shown. **(E–F)** Cell migration of control and FUT8-overexpressing 786-O and Caki-1 cells evaluated by Transwell assays (scale bar: 50 µm) **(E)** and wound-healing assays (scale bar: 50 µm) **(F)**, with quantification of migrated cells and wound closure. **(G–H)** In vivo tumor growth of FUT8-overexpressing cells. Representative images of subcutaneous xenograft tumors **(G)** and corresponding tumor growth curves **(H)** derived from 786-O cells expressing Vector or FUT8 OE (n = 5 per group). Data are presented as mean ± SD. Statistical significance was assessed using unpaired t tests or one-way ANOVA as appropriate. All in vitro experiments were performed in three independent biological replicates (n = 3). ns, P > 0.05; *P < 0.05; **P < 0.01; ***P < 0.001; ****P < 0.0001.


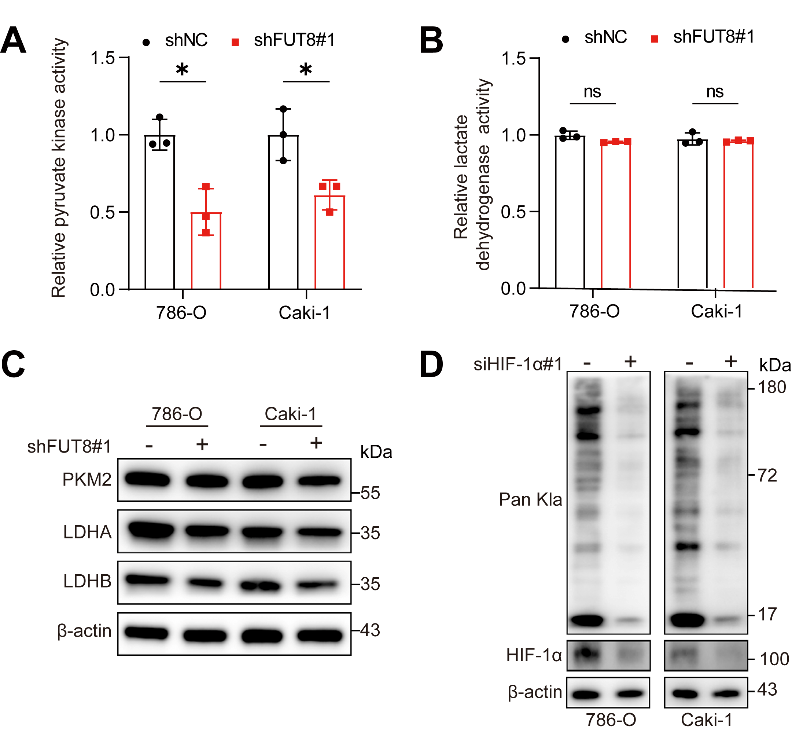


**Fig. S3 FUT8 knockdown reduces pyruvate kinase activity without affecting LDH activity and partially overlaps with HIF-1α–dependent lactylation. (A)** Pyruvate kinase (PK) activity in FUT8-knockdown and control 786-O and Caki-1 cells. **(B)** Lactate dehydrogenase (LDH) activity in FUT8-knockdown and control 786-O and Caki-1 cells. **(C)** Western blotting of PKM2, LDHA, and LDHB in FUT8-knockdown and control 786-O and Caki-1 cells. **(D)** Western blot analysis of global protein lysine lactylation (pan-Kla) and HIF-1α levels in 786-O and Caki-1 cells transfected with control or HIF-1α-targeting siRNA. Data are presented as mean ± SD. Statistical significance was assessed using unpaired t tests. All experiments were performed in three independent biological replicates (n = 3). ns, P > 0.05; *P < 0.05; **P < 0.01; ***P < 0.001; ****P < 0.0001.

**
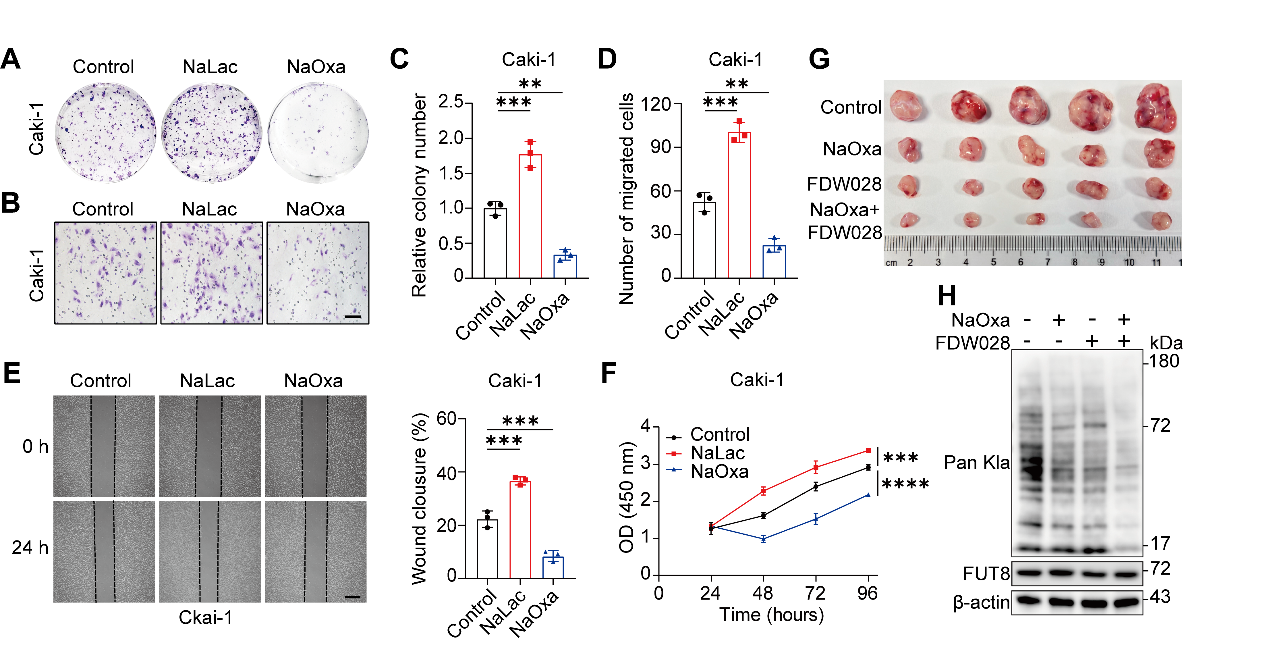
**

**Fig. S4 Lactate promotes malignant phenotypes in Caki-1 cells and provides representative data supporting Fig. 4M. (A, C)** Effects of lactate and lactate inhibition on cell proliferation in Caki-1 cells. Cells were treated with 20 mM sodium L-lactate (NaLac) or 10 mM sodium oxamate (NaOxa). Representative images **(A)** and quantification **(C)** of colony formation assays are shown. **(B, D)** Cell migration of Caki-1 cells under the indicated treatments assessed by Transwell assays (scale bar: 50 µm), with representative images **(B)** and quantification of migrated cells **(D)**. **(E)** Representative images and quantification of wound-healing assays performed in Caki-1 cells treated with Control, NaLac, or NaOxa (scale bar: 50 µm). **(F)** Cell proliferation of Caki-1 cells under the indicated treatments measured by CCK-8 assays. **(G)** Representative images of subcutaneous xenograft tumors derived from 786-O cells treated with Control, NaOxa, FDW028, or the combination of NaOxa and FDW028, corresponding to the in vivo data summarized in Fig. 4M. **(H)** Western blot analysis of pan-Kla and FUT8 expression in 786-O cells treated with the indicated condition. Data are presented as mean ± SD. Statistical significance was assessed using one-way ANOVA. All experiments were performed in three independent biological replicates (n = 3). ns, P > 0.05; *P < 0.05; **P < 0.01; ***P < 0.001; ****P < 0.0001.
