# Supplementary material for: FUT8 reprograms glycolytic metabolism to promote PKM2 lactylation and drive clear cell renal cell carcinoma progression
Source: Cell Death Discov. 2026 Mar 19;12:146. doi: 10.1038/s41420-026-03013-1 (PMC13039778; doi:10.1038/s41420-026-03013-1)
Supplement: Supplementary file 3 — Table S2 [file 41420_2026_3013_MOESM3_ESM.docx]

| **Gene** | **Sequences (5’-3’)** |
| --- | --- |
| HIF-1α-Forward | GAACGTCGAAAAGAAAAGTCTCG |
| HIF-1α-Reverse  FUT8-Forward  FUT8-Reverse | CCTTATCAAGATGCGAACTCACA  GAATCTCAGAATTGGCGCTATGC  GAAGCTCGACCACTTGAACAT |
| β-Actin-Forward  β-Actin-Reverse | CATGTACGTTGCTATCCAGGC  CTCCTTAATGTCACGCACGAT |

**1. RT-qPCR primers:**

**2. shRNA sequences:**

| **shRNA** | **Sequences (5’-3’)** |
| --- | --- |
| shFUT8#1 | CGTGGAGTGATCCTGGATATA |
| shFUT8#2 | CCACAGATGACCCTTCTTTAT |

**3. siRNA sequences:**

| **siRNA** | **Sequences (5’-3’)** |
| --- | --- |
| siHIF-1α#1 | GGAACATGATGGTTCACTT |
| siHIF-1α#2 | CTACCCACATACATAAAGA |
