# Supplementary material for: FUT8 reprograms glycolytic metabolism to promote PKM2 lactylation and drive clear cell renal cell carcinoma progression
Source: Cell Death Discov. 2026 Mar 19;12:146. doi: 10.1038/s41420-026-03013-1 (PMC13039778; doi:10.1038/s41420-026-03013-1)

Fig. 1D

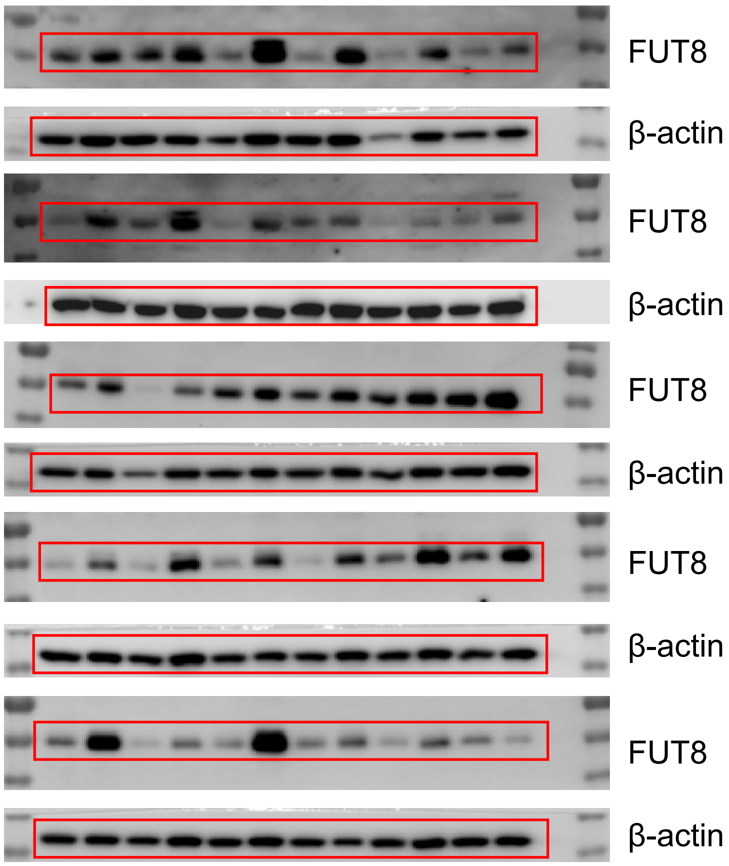

Fig. 2A

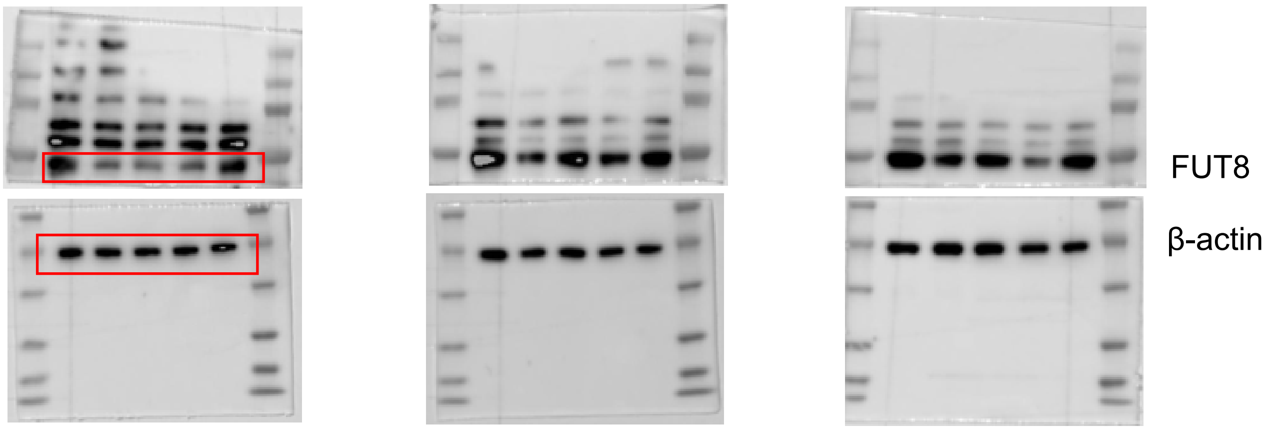

Fig. 2J

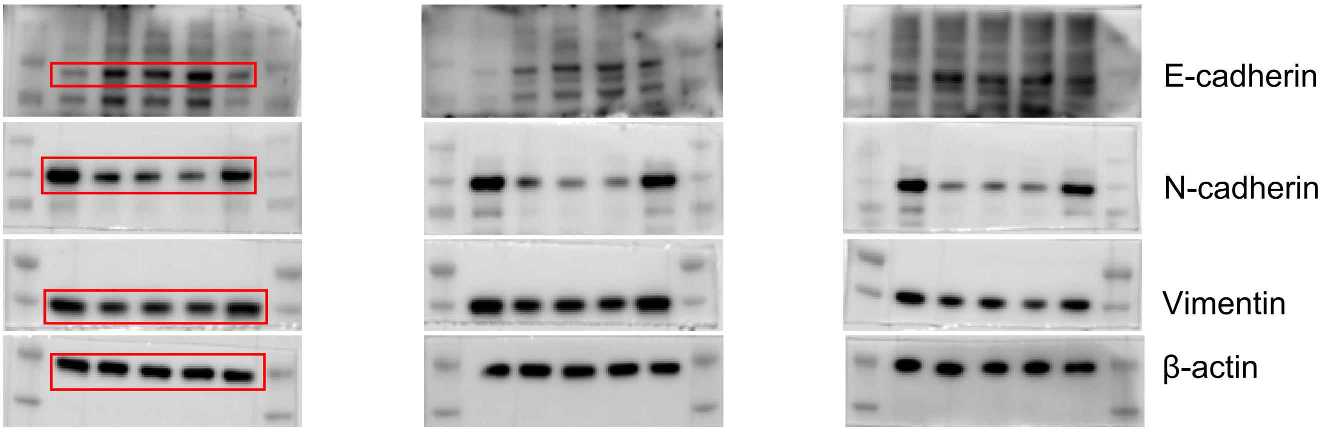

Fig. 3D

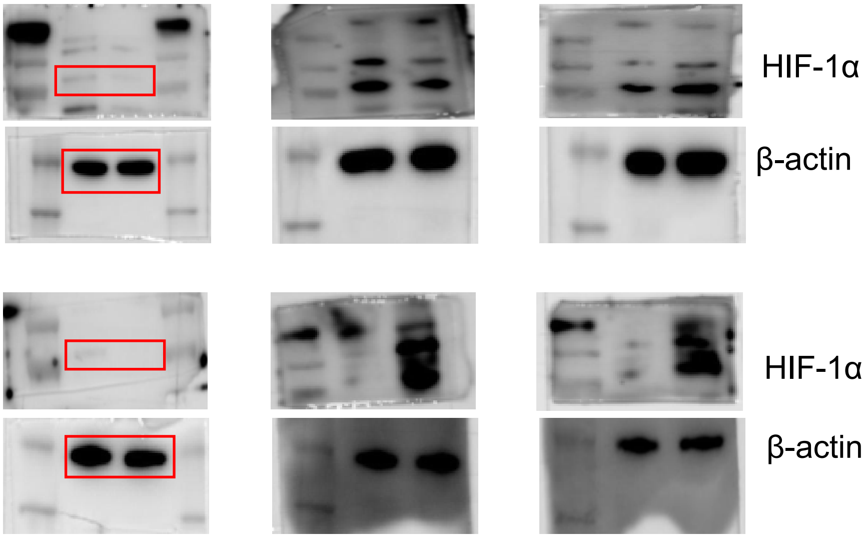

Fig. 3K

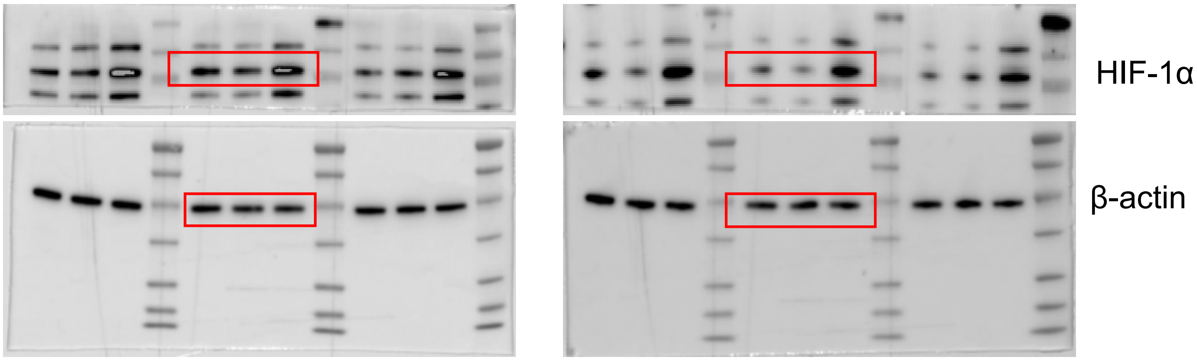

Fig. 5A

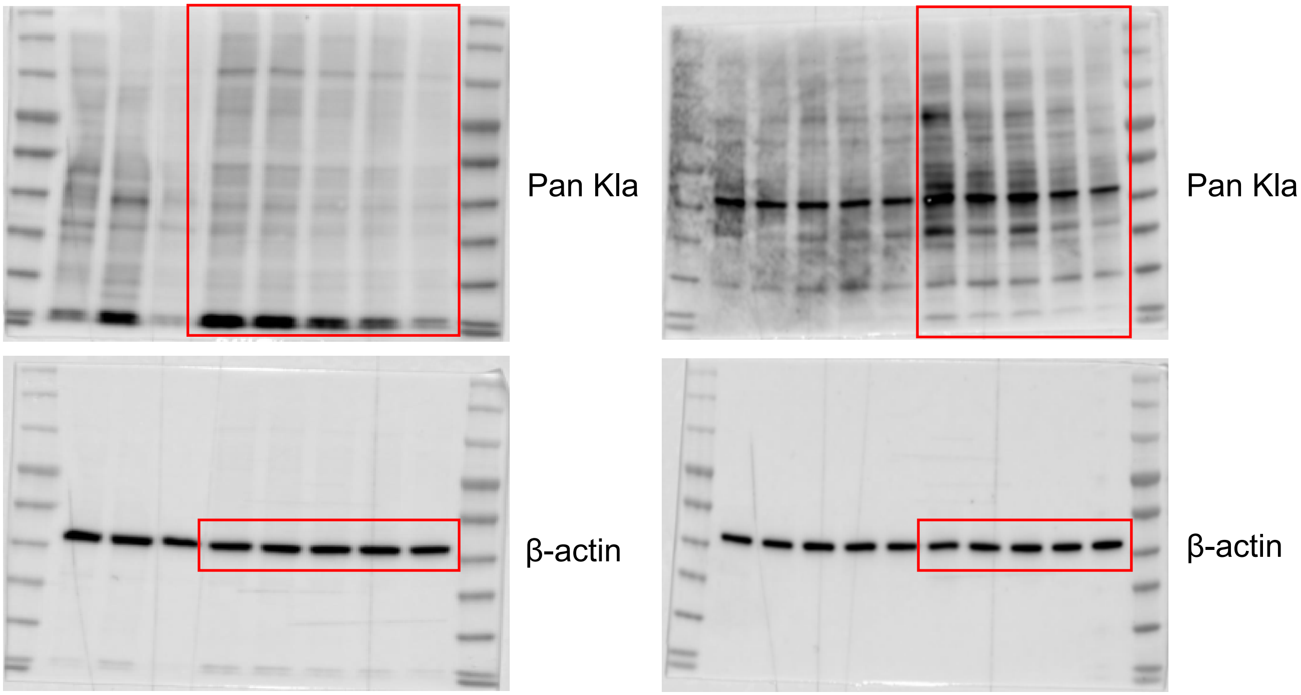

Fig. 5B

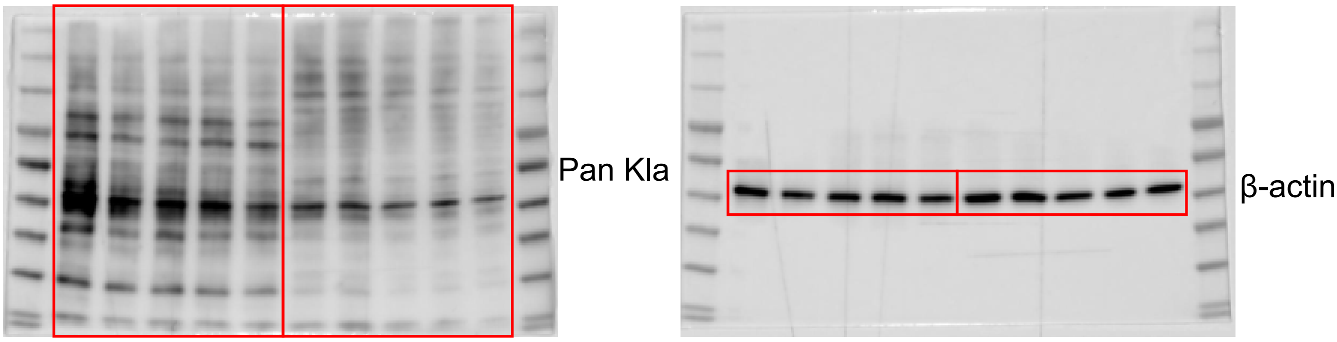

Fig. 5C

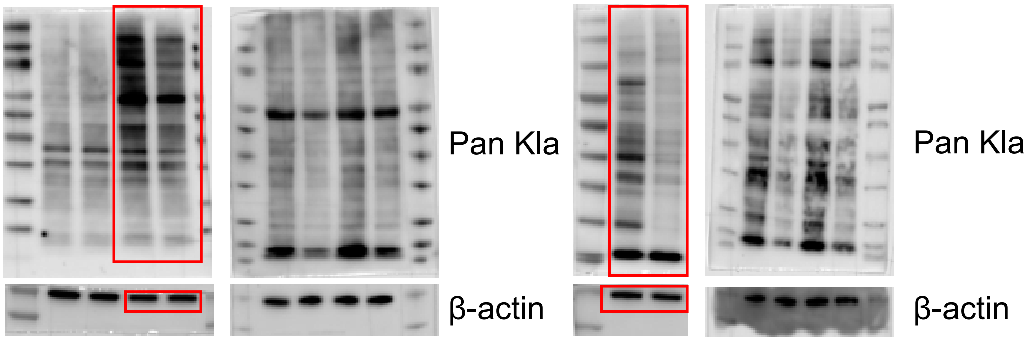

Fig. 5D

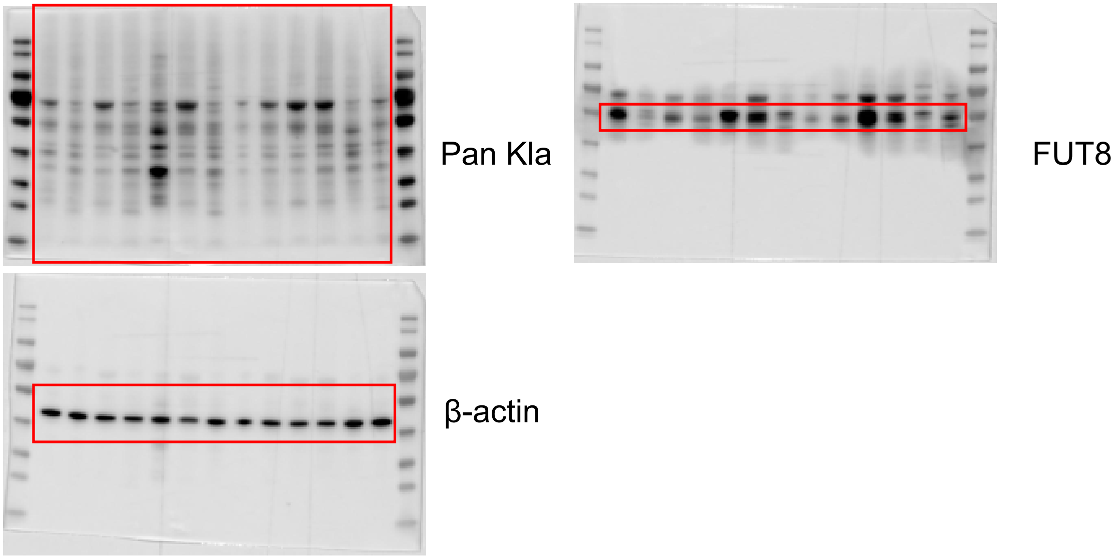

Fig. 5J

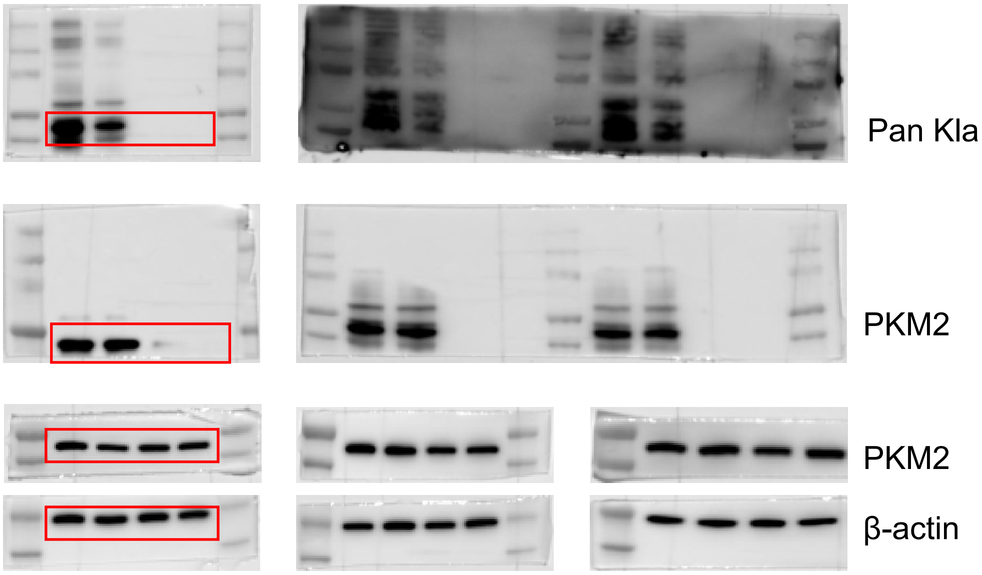

Fig. 6A

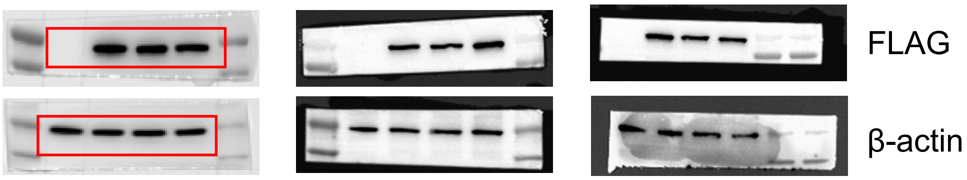

Fig. 6J

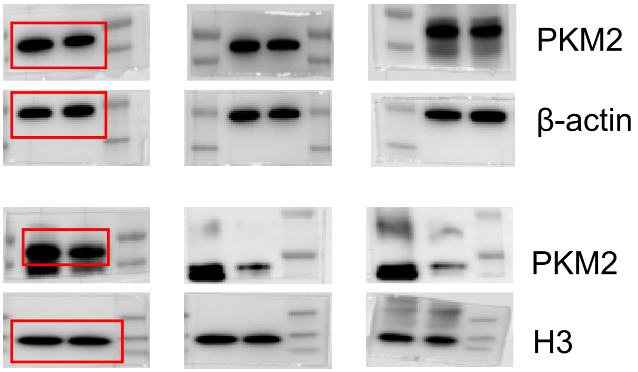

Fig. 6H

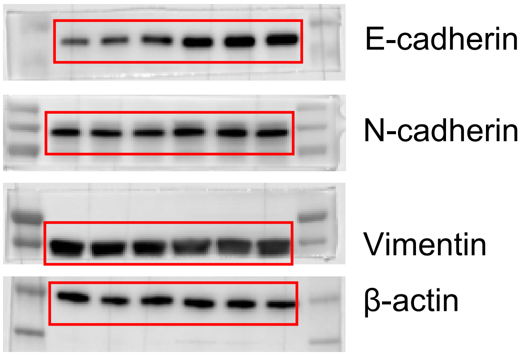

Fig. S1A

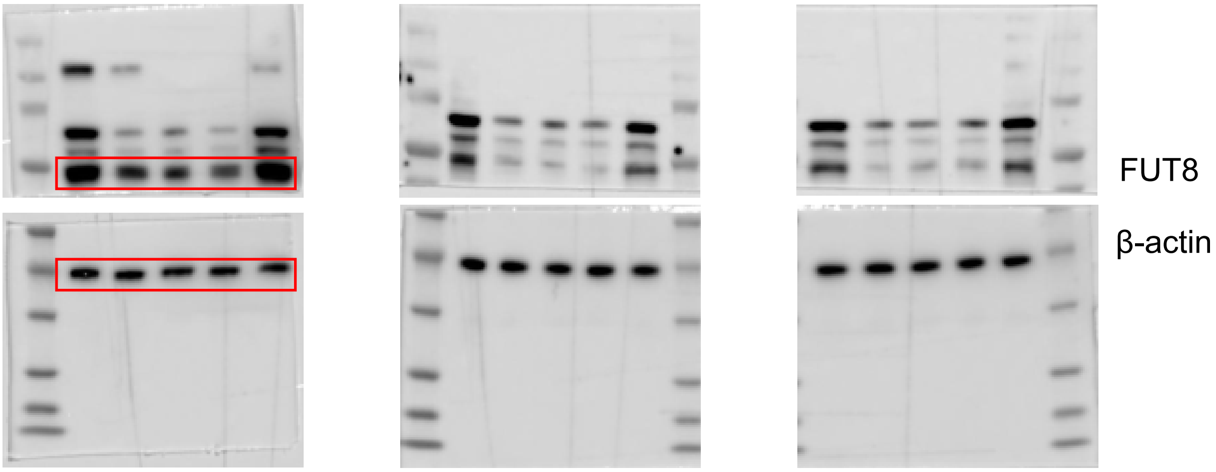

Fig. S1J

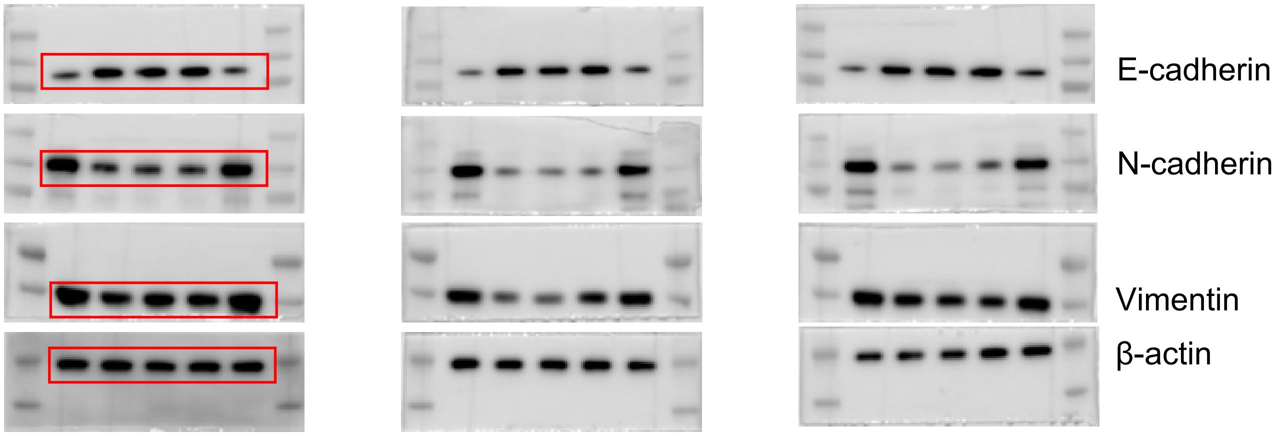

Fig. S2A

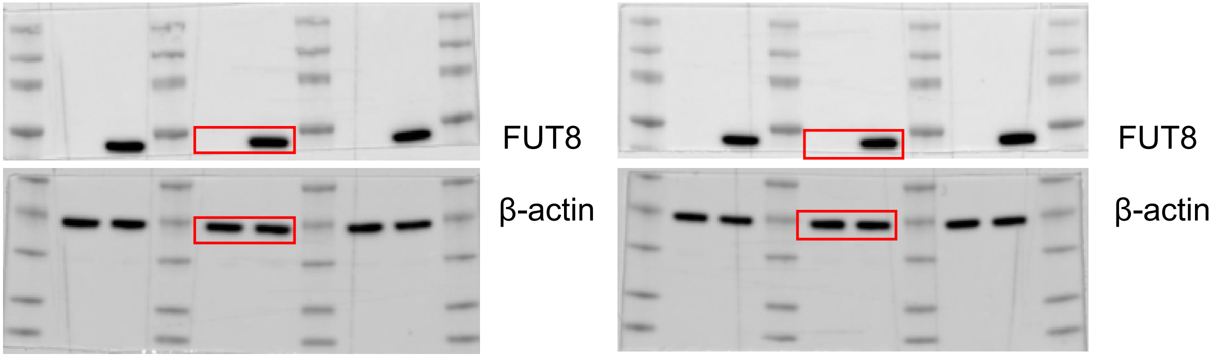

Fig. S3D

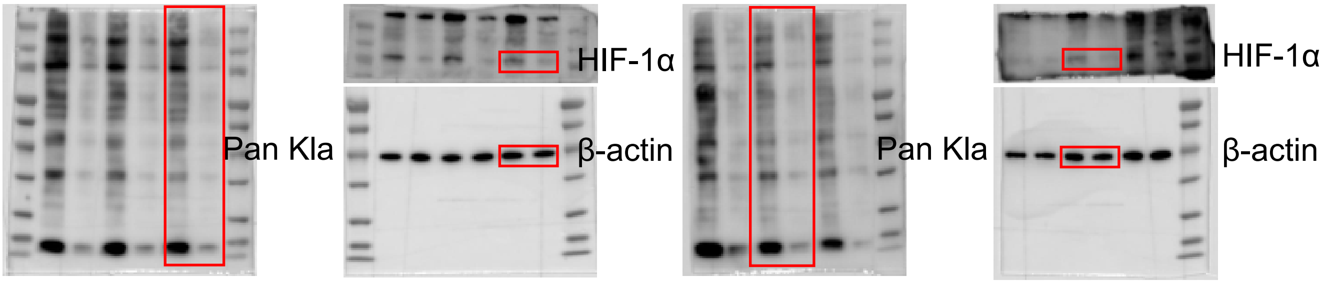

Fig. S3C

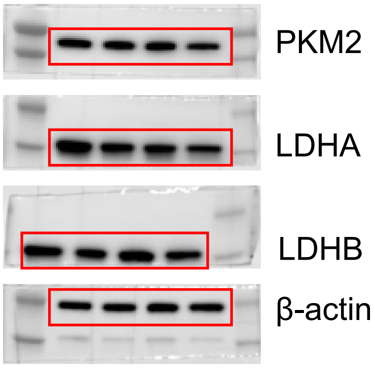

Fig. S4H

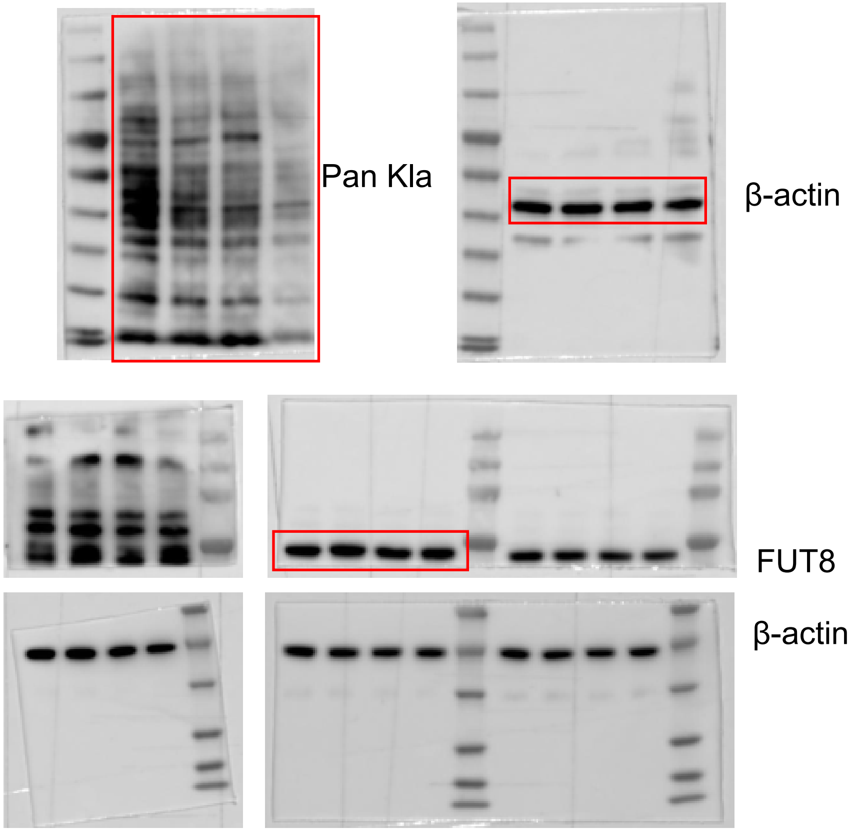

Supplement: Supplementary file 6 — Original western blots [file 41420_2026_3013_MOESM6_ESM.pdf]
